# Identification of host cell surface proteins inhibiting furin dependent proteolytic processing of viral glycoproteins

**DOI:** 10.1038/s41598-025-11164-x

**Published:** 2025-07-15

**Authors:** Nathalia Williams, Mehdi Chabert, Alicia Besomi, Filo Silva, Karolina Sobiech, Mirco Schmolke

**Affiliations:** 1 Department of Microbiology and Molecular Medicine, Faculty of Medicine, University of Geneva, Geneva, Switzerland; 2 Geneva Center for Inflammation Research, University of Geneva, Geneva, Switzerland

**Keywords:** Influenza virus, SARS-CoV-2, Virus-host interactions

## Abstract

Proteolytic cleavage by furin-like proteases is a crucial first step in the posttranslational modification of various glycoproteins found in enveloped emerging viruses, such as SARS-CoV-2 and highly pathogenic avian influenza A viruses (IAV). Here, we explored the capacity of host cell proteins identified by cell surface proximity ligation to limit the proteolytic cleavage of the SARS-CoV-2 spike and the IAV H5N1 hemagglutinin (HA). When co-expressed with recombinant SARS-CoV-2 spike protein, Prom1, Axl, and Ly75 suppress its proteolytic cleavage, whereas cleavage of HA was only reduced by Prom1. Co-immunoprecipitation assays suggest that Axl and Prom1 may form a complex with furin. Alteration of Prom1, Axl and Ly75 expression levels in Calu3 cells affected entry of SARS-CoV-2 S pseudotyped VLP and to a lesser extent, SARS-CoV-2 virions. In contrast, Prom1 levels did not affect entry of H5N1 VLPs or H5N1 virions. Our data highlight the differential capacity of SARS-CoV-2 and IAV H5N1 to cope with newly identified host restriction factors of furin activity.

## Introduction

Enveloped viruses display proteins on their surface that are essential for receptor engagement, fusion with the host membrane and subsequent entry into the host cell. To achieve a fusion-ready confirmation, post-translational proteolytic cleavage of these viral ligands by host endo-proteases is a prerequisite for many human pathogenic enveloped viruses^1^. Amongst these are also emerging viruses, e.g. SARS-CoV-2^2,3^ or highly pathogenic H5N1 avian IAV.

SARS-CoV-2 infections in humans were first described in late 2019 in Wuhan China, probably as a consequence of a zoonotic transmission event on a live animal market^4^. Since then, they caused a global pandemic with more than 7 Mio confirmed deaths^5^. SARS-CoV-2 virions attach to heparan sulfates on the cell surface and rely primarily on angiotensin converting enzyme 2 (ACE2) to enter the host cell^2^, although ACE2 independent entry was reported^6^. The viral spike (S) protein mediates this process by binding to ACE2 through its receptor-binding domain, initiating the entry process^7^. After receptor engagement, the fusion peptide is exposed facilitating the fusion of viral and cellular membranes—either at the plasma membrane or within the endosomal membrane. The entry process requires proteolytic activation of the SARS-CoV-2 Spike (SARS-CoV-2 S). The multi-basic cleavage site (MCS) of SARS-CoV-2 S is processed by furin-like proteases prior to virion assembly in the Golgi apparatus of the virion producing cell or in the extracellular space by secreted furin-like proteases^7^.

A second cleavage step occurs either at the cell surface via the serine protease TMPRSS2 or during endosomal uptake by pH-sensitive cathepsins^2^. This second cleavage event releases the fusion peptide, initiating membrane fusion. Early SARS-CoV-2 isolates predominantly fused at the cell surface, while the more recent Omicron strains preferentially enter via the endosomal route.

Furin is a trans golgi network (TGN) resident, membrane-anchored member of the proprotein convertase (PC) family of calcium-dependent serine endoproteases. Due to a sorting signal in its cytoplasmic tail, furin can shuttle from the TGN to the cell surface where it is eventually released into the extracellular space after proteolytic cleavage. Genetic knockout of furin leads to embryonic death in mice, underlining the importance of this proprotein convertase in early development (reviewed in^3^). Targeting furin has been proposed as a strategy to inhibit SARS-CoV-2 infections^8^.

Similarly, the entry of other enveloped viruses, such as highly pathogenic avian influenza A (H5N1), also depends on precise glycoprotein cleavage and membrane fusion events^9^. A highly pathogenic avian IAV of the H5N1 subtype has been a major concern for human health since the first direct transmission events from infected poultry to humans were detected^10^. While these events are sporadic and do not lead to consecutive human to human infections beyond small family clusters, the high mortality rate and the potential of this virus family to adapt to mammalian host spurred concerns. The recent outbreaks of avian origin H5N1 viruses in mink farms^11^ and in milking cows^12^ underline this threat as well as reports of transmissions to humans from the latter^13,14^.

We employed here cell surface proximity ligation assay (CSPL)^15^ to label host plasma membrane proteins in the vicinity of an attached viral S protein with the aim of identifying host regulators of proteases required for viral entry.

## Results

**Fig. 1:**
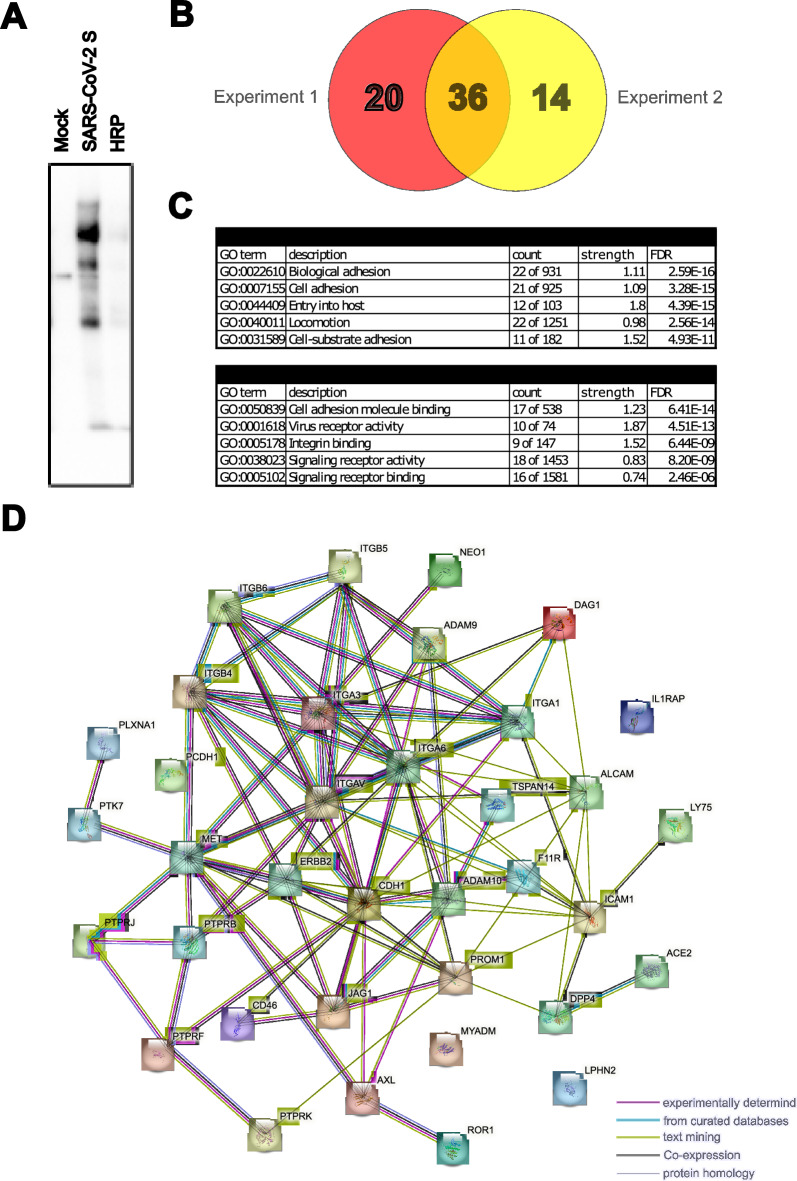
Cell surface proximity ligation. (**A**) Biotinylated proteins were pulled down with streptavidin beads on Calu3 cells post cell surface proximity ligation. Proteins were probed with a streptavidin-HRP. (**B**) Venn diagram indicating number of proteins found in the individual and the overlap between the two Calu3 mass spectrometry experiments. (**C**) GO cluster of the identified proteins to indicate their function in viral receptor and entry. Count refers to the number of proteins annotated in a particular network over the total number of proteins in this network. Strength (indicator of enrichment) indicates the ratio of proteins from the network assigned to a term over the expected number of proteins assigned to a random network of equal size. (**D**) String network of the 36 proteins found in the two mass spectrometry experiments.

In a first step we performed CSPL as previously described^16^ on Calu3 cells using a trimeric SARS-CoV-2 S ectodomain fused to HRP as a molecular bait (Fig. S1). These cells are naturally permissive for SARS-CoV-2 and express both ACE2 and TMPRSS2^17^. Biotinylated proteins were pulled down with streptavidin beads (Fig. 1A) and analyzed by mass spectrometry after on-bead proteolysis with trypsin.

**Fig. 2:**
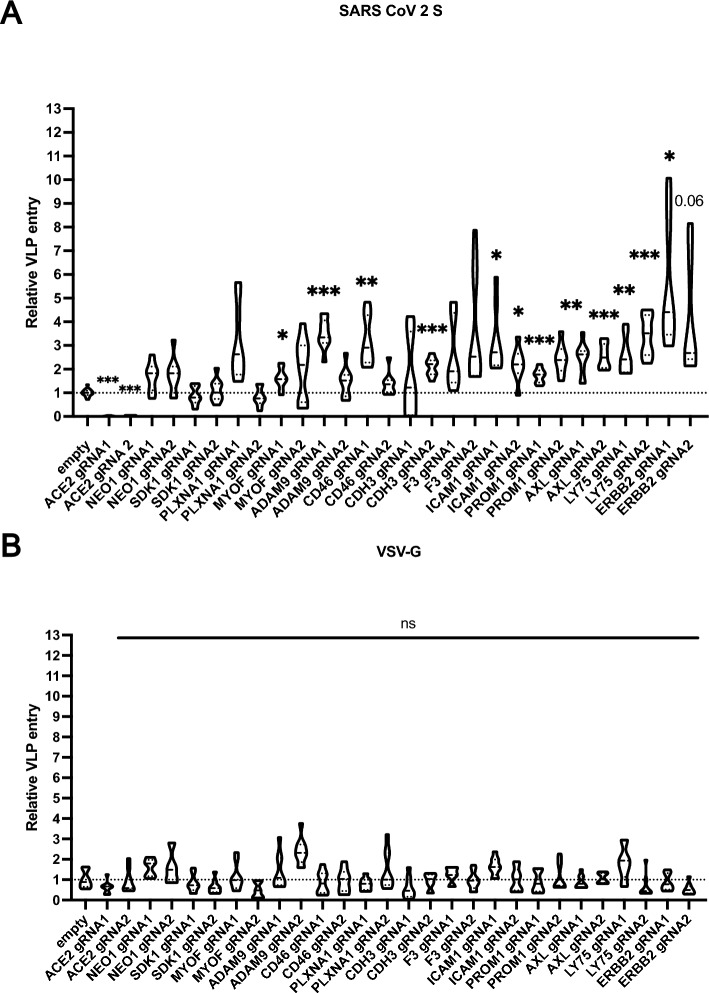
SARS-CoV-2 S and VSV-G pseudotyped VLP entry into Calu3 KO cells. (**A**) SARS-CoV-2 pseudotyped VLP entry in Calu3 cells transduced with lentiviral CRISPR/Cas9 guide RNAs (2 per target). ACE2 was used as a positive control. (**B**) VSV-G pseudotyped VLP entry in Calu3 cells transduced with lentiviral CRISPR/Cas9 guide RNAs (2 per target). The results are shown in fold change to control in relative light units. *P* values were calculated with Brown Forsythe and Welch ANOVA (* < 0.05, ** < 0.01, *** < 0.001).

In total, 36 proteins were shortlisted after bioinformatic filtering across the two independent CSPL experiments (Fig. 1B and Table S1). Among these were known proteins involved in SARS-CoV-2 entry, such as the *bona fide* entry receptor ACE2. STRING database network^18^ analysis of the identified proteins showed enrichment for the GO clusters “Entry into host cell” (Biological process) and “virus receptor activity” (Molecular function) with false discovery rates of 10^−15^ or 10^−13^ respectively (Fig. 1C). In contrast, proteins identified in only one of the two mass spec data sets (Fig. S2A) were not associated with the biological process “Entry into host cell” and the molecular function “virus receptor activity” had a false discovery rate of 10^−7^ (Fig. S2B). Some target proteins (e.g. ITGAV, ITGB4 or ALCAM) were previously tested in a screen for proviral host factors of SARS-CoV-2 entry in CSPL study on A549 cells overexpressing ACE2 and TMPRSS2^16^ and hence omitted here. We proceeded with a subset of the 36 preliminary targets and generated knockout Calu3 cell lines for 13 targets using 2 gRNAs for each target gene, which were transduced into Calu3 using lentiviral vectors followed by antibiotic selection in bulk. Using HIV based virus like particles (VLP) encoding for a Gaussia luciferase reporter gene, we tested the effect of each knockout cell line for SARS-CoV-2-S (Wuhan) or VSV-G mediated viral entry (Fig. 2A, B). Knockout was confirmed by specific Western blot (Fig. S3). Only 6 targets showed significant changes in VLP entry (ACE2, LY75, AXL, ICAM1, ERBB2, PROM1) with both gRNAs (Fig. 2A). We excluded those targets where VLP entry was not affected despite successful knockout in one (ADAM9, MYOF, CD46, CDH3) or both gRNAs (SDK1, F3, PLXNA1). The knockout for NEO1 could not be confirmed by Western blot and ILRAP1 knockouts were not viable, hence these targets was dropped. Notably, with exception to the knockout of the bona fide entry receptor ACE2 all remaining knockout cell lines displayed enhanced SARS-CoV-2 S dependent VLP entry, suggesting that the respective target proteins have a restrictive function on the viral entry process. Importantly, entry of VSV-G pseudotyped VLP was not affected in any of knockout cell lines (Fig. 2B), supporting a SARS-CoV-2 S specific phenotype.

**Fig. 3:**
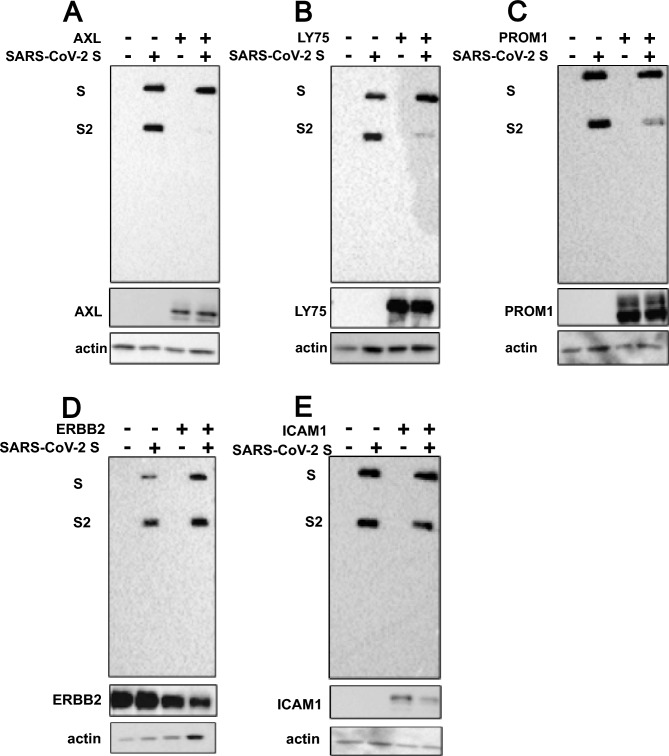
Effect of target proteins on spike cleavage. Cell lysates were separated by SDS-PAGE and analyzed by western blot depicting the expression of target proteins and SARS-CoV-2 S (both the full-length spike, S and the cleaved spike subunit, S2). The lysates were obtained post-transfection of a spike plasmid (pTwist-EF1α-nCoV2019-S-2xStrep) and the cDNA plasmids of the target proteins in HEK 293T cells depicted from A–D. The blots are indicated for the presence and absence of the different plasmids by – and + signs. Co-transfection experiments with AXL, LY75, PROM1, ERBB2 and ICAM1 are shown with one representative out of three experiments. Equal loading was confirmed by probing for beta actin.

**Fig. 4:**
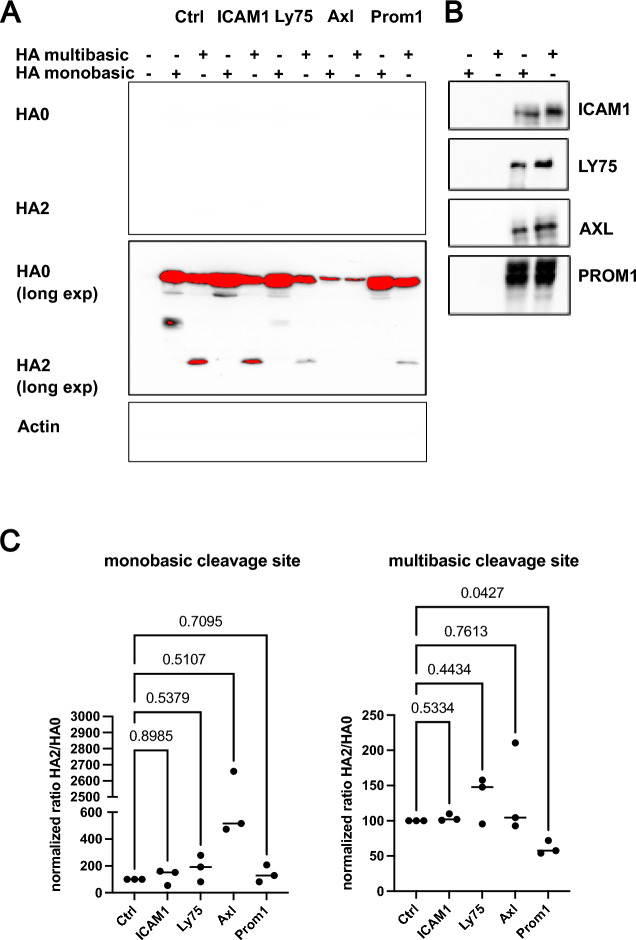
Effect of target proteins on H5N1 HA cleavage. (**A**) Cell lysates of transfected HEK 293T cells were separated by SDS-PAGE and analyzed by western blot for HA0 (uncleaved) and HA2 (cleaved) via a C-terminal flag epitope tag. A HA variant with monobasic or multibasic cleavage site were co-expressed with indicated host proteins in HEK 293T cells. One representative blot out of three experiments is shown. Equal loading was confirmed by probing for beta actin. (**B**) Western blot control for overexpression of the 4 restriction factors tested in panel (**A**). (**C**) Quantification of band intensity from three independent coexpression experiments as depicted in panel A. Values are normalized to the respective control condition for HA with monobasic (left) or multibasic (right) cleavage site. Statistical significance was determined by multiple one-way ANOVA testing. *P* values for indicated sample pairs are provided.

To assess an involvement of the shortlisted proteins, on proteolytic cleavage of SARS-CoV-2 S we co-expressed it with LY75, AXL, ICAM1, ERBB2 or PROM1 in 293T cells. Western blot analysis revealed that three (Axl, Prom1 and Ly75) out of the five proteins, clearly reduced the S2 band, suggesting a repressive effect on proteolytic processing (Fig. 3A–C). There was no major effect on the full-length spike, excluding a general degradation during co-expression. ICAM1 and ERBB2 expression had no effect on S protein processing (Fig. 3D, E). SARS-CoV-2 is not the only virus that depends on furin-dependent cleavage of its viral ligand for entry (reviewed in^19^). To test if Axl, Prom1 and Ly75 would also prevent cleavage of other viral glycoproteins, we representatively tested the hemagglutinin (HA) of highly pathogenic avian influenza A virus (A/Vietnam/1203/2004 (H5N1)). Furin cleaves the proprotein HA0 into HA1 and HA2. HA was C-terminally fused to a flag-epitope, allowing the detection of HA0 and HA2 by anti-flag antibodies and the detection of HA0 and HA1 by a H5 specific HA head domain-specific antibody. Surprisingly, only the co-expression of Prom1 with the HA of H5N1, reduced the relative amount of cleaved HA2 with a multibasic cleavage site (Fig. 4A–C (right panel)). In absence of trypsin the HA with the monobasic cleavage site remained uncleaved (Fig. 4C (left panel)). Unexpectedly, Axl co-expression abolition HA expression completely, an effect for which we currently have no explanation.

**Fig. 5:**
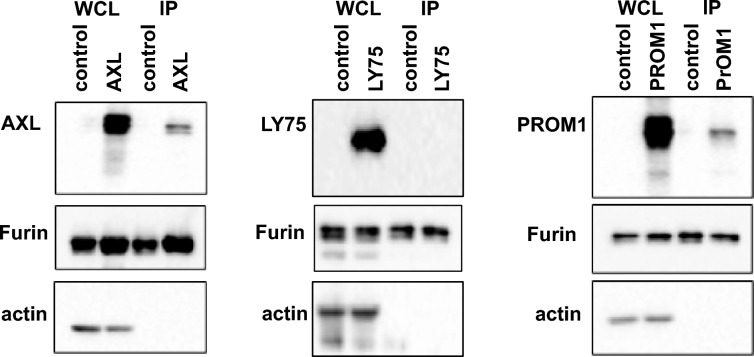
Co-immunoprecipitation of furin with the target proteins. Cell lysates were obtained post co-transfection of the human furin plasmid with the cDNAs of AXL, LY75 or PROM1 in 293T cells (Whole cell lysate, WCL) and also post immunoprecipitation with flag beads that bind to the human furin plasmid to pull down interacting partners (IP). The lysates were separated on SDS-PAGE and analyzed by western blot depicting the expression of target proteins and human furin. Beta-actin was used as a loading control for the WCL. One representative of three experiments is shown for AXL, LY75 and PROM1.

Next, we investigated whether the effect of Axl, Prom1 and Ly75 on proteolytic cleavage of SARS-CoV-1 spike depends on a physical interaction with furin. Co-immunoprecipitation experiments revealed complex formation between Axl and Prom1 with furin, while Ly75 did not co-immunoprecipitate with furin (Fig. 5), suggesting either an alternative more indirect mode of action for Ly75, or a generally weaker interaction preventing successful coprecipitation. We hence decided to concentrate on Axl1 and Prom1 for the mechanistic analysis.

**Fig. 6:**
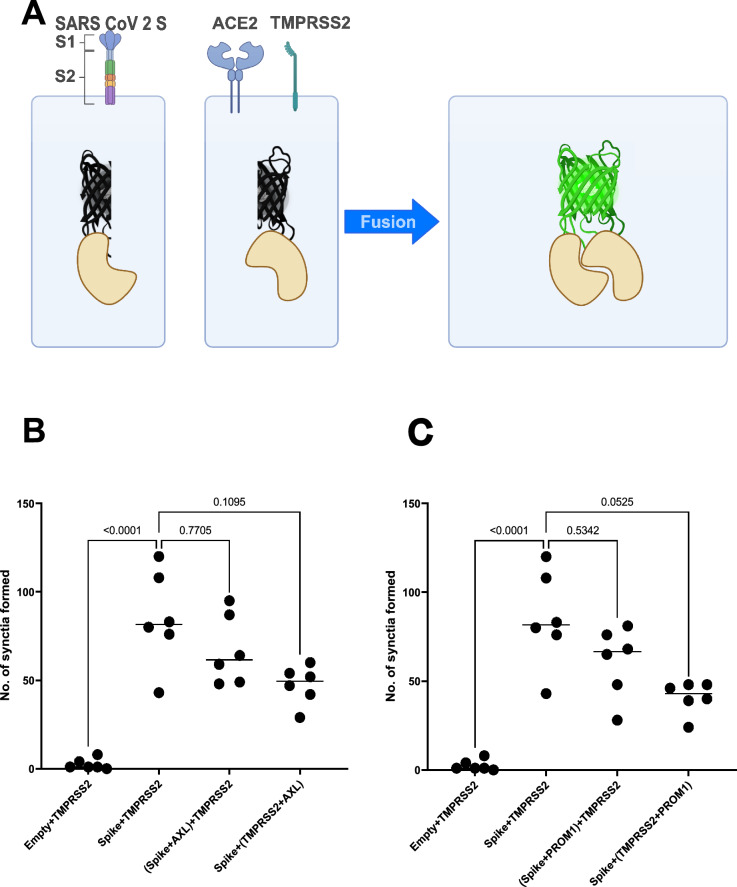
Bimolecular fluorescence complementation assay (BiFC) to study syncytium formation. (**A**) Schematic representation of the BiFC assay. VeroE6 cells expressing spike protein in the presence of a monomer of the GST and on the other side Vero E6 cells expressing TMPRSS2 and ACE2 proteins with the other monomer of the GST protein. These cells combined will result in the formation of multi-nucleated cells referred to as syncytia which can be visualized by GFP signal using ImageXpress microscope. (**B**) The BiFC experiment was performed with cells expressing AXL, along with the spike protein or with the TMPRSS2 and ACE2 (shown in Table 1). Two biological replicates are shown with triplicates per biological replicate. One-way ANOVA was performed as a statistical test. (**C**) The BiFC experiment was performed with cells expressing PROM1, along with the spike protein or with the TMPRSS2 and ACE2 (shown in Table 1). Two biological replicates are shown with triplicates per biological replicate. One-way ANOVA was performed as a statistical test.

Furin cleavage of target proteins occurs both in the TGN and the extracellular space, as the proprotein convertase is actively secreted^20^. Therefore, the direct inhibition of furin dependent processing of SARS-CoV-2 S by Prom1 and Axl could theoretically occur either intra-cellularly in the TGN of virion-producing cells or in the extracellular space on the surface of virion-targeted cells. The latter scenario aligns with the results from the proximity ligation experiments (Fig. 1). To experimentally test whether the two furin binding inhibitors Axl and Prom1 act on the virus-producing or virus-targeted cell, we setup a SARS-CoV-2 S dependent cell–cell-fusion-assay. We used a split-YFP system fused to glutathione S transferase (GST), where GST dimerizes and brings the two halves of YFP into proximity, enabling the formation of a functional fluorophore^21^. We expressed GST YFP-N and GST YFP-C in separate cell batches of Vero E6 cells and mixed them after transfection (Fig. 6A). No or very low fluorescence signal was observed under these conditions (Fig. 6B, C). Co-expression of SARS-CoV-2 S on the donor cells and TMPRSS2 on the acceptor cells resulted in syncytia formation with YFP-complexes, since Vero E6 endogenously express ACE2, which serves as the main receptor for SARS-CoV-2^22^. The introduction of Axl or Prom1 diminished cell fusion, but only when expressed on the acceptor cell side (Fig. 6B, C). These data suggest that Prom1 and Axl interfere with furin-dependent cleavage of SARS-CoV-2 S at the surface of target cells. To functionally link reduced SARS-CoV-2 S dependent VLP entry with the inhibition of furin activity we repeated the entry assays using VLPs pseudotyped with WT S (multibasic cleavage site) or a monobasic variant. To achieve comparable entry conditions with the two S variants, we had to adjust the readout time to 48 h post infection. Knockout of LY75, PROM1 or AXL enhanced only entry of VLPs decorated with SARS-CoV-2 S bearing a multibasic (furin dependent) cleavage site (Fig. S4).

**Fig. 7:**
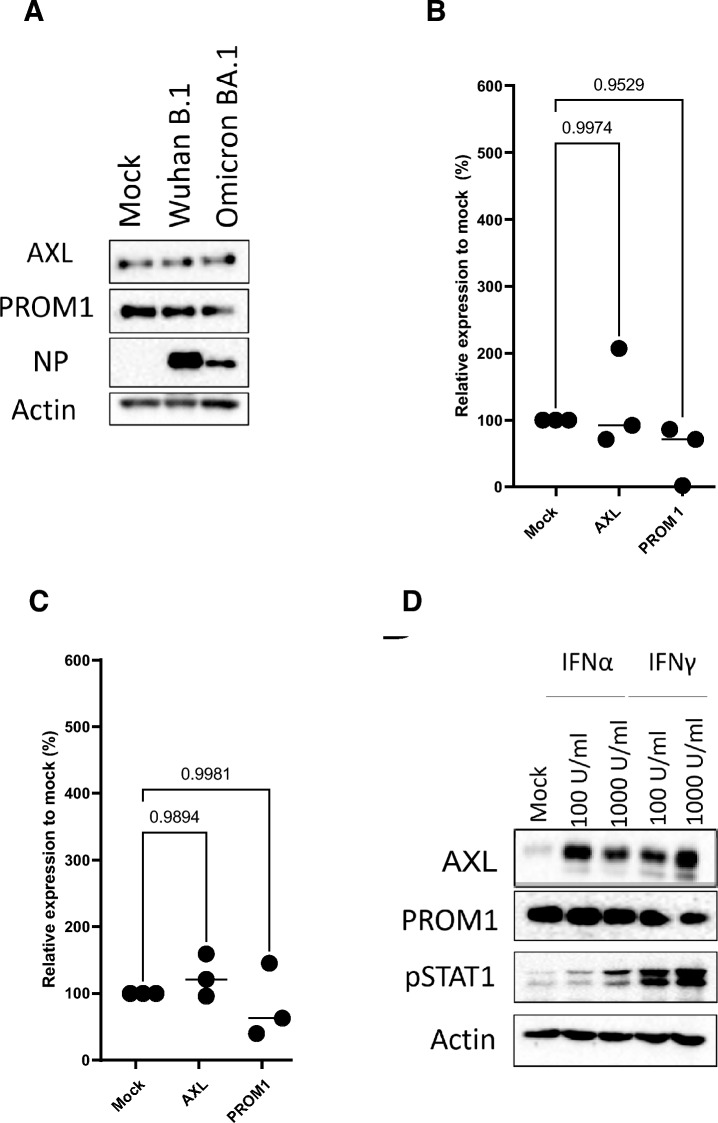
Interferon and SARS-CoV-2 infection regulate target protein expression. (**A**) Wild-type Calu3 cells were treated with Interferon α and γ at different concentrations (100 U/ml and 1000 U/ml), for 24h and cell lysates were separated by SDS-PAGE and analyzed by western blot. The target protein expressions are depicted PROM1 and AXL. Beta-actin was probed as a loading control. Phosphorylation of STAT1 (pSTAT1) is also indicated to verify interferon activity. (**B**) Cell lysates were separated by SDS-PAGE and analyzed by western blot to show the expression of the target proteins 24 h post infection with Wuhan B.1 or Omicron BA.1 at an MOI of 1. The SARS-CoV-2 N protein was probed to verify infection and beta-actin was probed for loading control. A representative of three experiments is shown. (**C**) Quantification of target proteins as shown in (**A**) in Calu3 cells treated with Interferon α and γ at different concentrations for 24 h. Three biological replicates are shown in the graph. One-Way ANOVA statistical test was performed to compare the Calu3 control cells with the target proteins. (**D**) Quantification of target proteins as shown in (**B**) in Calu3 cells infected with Wuhan B.1 and Omicron BA.1 at an MOI of 1 for 24 h. Three biological replicates are shown in the graph. One-Way ANOVA statistical test was performed to compare the Calu3 control cells with the target proteins.

We were interested in whether the expression levels these surface proteins change following SARS-CoV-2 infection. At 24 h post-infection with either Wuhan or Omicron strains, the levels of AXL and PROM1 were unaffected (Fig. 7A–C). SARS-CoV-2 infection provokes a robust innate host response in the respiratory tract of infected hosts^1^. To assess if key cytokines of the antiviral response might trigger upregulation of the newly identified HRF we chose to stimulate Calu3 cells with type I and type II interferon and measure the expression levels of here identified host restriction factors by western blot (Fig. 7D). Type II interferon induced a robust upregulation of Axl while the same treatment had no effect on Prom1 expression. In contrast, the concentrations of type-I interferon used had no effect on the expression of the host restriction factors. These data suggest that SARS-CoV-2 infection upregulates certain negative regulators of furin, with type II interferon playing a potential role in modulating this response in vivo and suppressing furin activity.

**Fig. 8:**
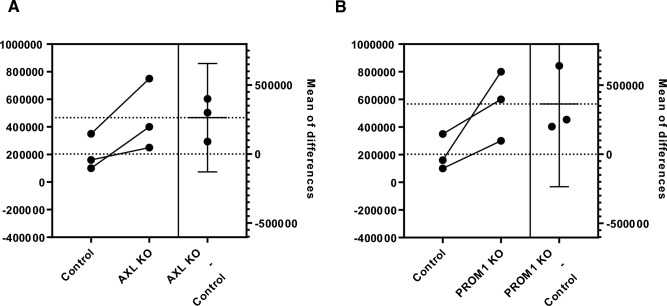
Omicron BA.1 virus replication in Calu3 KO cells. (**A**) Plaque assay results between the Control and the AXL KO cells 72 h post infection with Omicron BA.1 at an MOI of 1 are shown. (**B**) Plaque assay results between the Control and the PROM1 KO cells 72 h post infection with Omicron BA.1 at an MOI of 1 are shown. A paired t-test was performed as a statistical test.

Lastly, we assessed whether genetic depletion of AXL and PROM1 would impact replication of furin dependent viruses in cell culture. For SARS-CoV-2 factors affected viral titers at early time points post infection, although to a rather modest level (Fig. 8). To see if the effect of Prom1 on HA cleavage would affect H5N1 HPAIV entry, we used BlaM1 VLPs pseudotyped with H5 HA containing either a polybasic or monobasic cleavage site. However, we observed no consistent effect of Prom1 expression on H5N1 IAV entry, regardless of the cleavage site type (Fig. S5A). Consistently, no Prom1-dependent growth phenotype for H5N1 HPAIV or the respective low pathogenic mutant virus expressing an HA with a monobasic cleavage site was observed (Fig. S5B, C).

## Discussion

Since the early days of the SARS-CoV-2 pandemic, it became clear that host susceptibility to infection and the severity of COVID-19 are influenced by genetic predisposition^23^. In this context, numerous host susceptibility factors have been identified. In contrast the underlying mechanisms driving intrinsic resistance to SARS-CoV-2 infection is more challenging to pinpoint. Early ISG focused screens identified DAXX and BST2/tetherin as cellular restriction factors for SARS-CoV-2^24,25^. Importantly, SARS-CoV-2 has evolved an evasion mechanism against DAXX, demonstrating the selection pressure elicited by this restriction factor. Among the tested ISGs, Ly6E a protein shown earlier in two studies to inhibit entry of SARS-CoV-2 and other coronaviruses into the host cell was identified^26,27^. In this study, we pursued an approach based on the proximity of host proteins to attached viral ligand. This assay does not rely on an immediate functional readout, which could explain the relatively high background of host surface proteins with no direct involvement in virus entry. Similarly, we identified proviral entry factors for influenza A and SARS-CoV-2 virus like this^15,17^. Among the identified host restriction factors with a robust effect on SARS-CoV-2 entry, only Axl and Prom1 acted directly by interfering with the activity of spike-activating proteases (Ly75 provided a similar effect however a direct interaction was not confirmed). LY75, Axl and Prom1 are expressed in human respiratory tissue, referencing the human protein atlas^28,29^. Ly75 is expressed at moderate levels throughout the respiratory tract, Axl is expressed at moderate levels in alveolar epithelial cells and macrophages and Prom1 is expressed in high levels in ciliated cells of the bronchi^28,29^.

ICAM1 and ERBB2 did not alter SARS-CoV-2 S cleavage suggesting alternative mechanisms that future studies will need to explore. Within the respiratory tract, ERBB2 is mostly expressed in epithelial cells of the nasopharynx and the lung and to a lesser extend in the bronchii. It is a member of the epithelial growth factor receptor family of plasma membrane localized tyrosine kinases. In contradiction to our findings, a very recent study showed that chemical inhibitors of this receptor family block SARS-CoV-2 entry^30^. ICAM1 serves as a polio virus^31^ and rhinovirus receptor^32^.

Interestingly, Axl was previously described as a proviral entry factor for SARS-CoV-2^33^. A similar contradiction was previously reported for IFITM3, which acts as restriction factor for SARS-CoV-2 fusion in the endosome. Consequently, overexpression of TMPRSS2 (biases the virus to enter at the cell surface) alleviates the effect of IFITM3 expression^34^. Another surface proximity ligation approach using the RBD of spike also identified Prom1^35^, with follow up experiments in 293T overexpressing ACE2 assigning a proviral function to this ISG. The bias towards endosomal entry, might explain this phenotype, alongside the inhibitor function of Prom1 in Calu3 cells. Notably, Prom1 was recently predicted by a machine learning approach to associate with furin^36^. These findings highlight the importance of the cellular context in which infection occurs. While we are confident that Calu-3 serve as a good cell line model for SARS-CoV-2, the bias towards cell surface entry may not be representative of all host cells in the human respiratory tract. The restriction of HA (H5 with multibasic cleavage site) cleavage by Prom1 did not translate into significant alterations in viral entry or growth, suggesting that alternative proteases could substitute for furin or that the Prom1 mediated inhibition is insufficient to fully block proteolytic activation.

Other host proteins have been shown to impact furin activity, such as Serpin B8, MARCH8, GBP2/5 alpha SNAP^37^ among others (reviewed in^38^). The impact of many of these factors on SARS-CoV-2 or H5N1 entry remains unknown. Redundancy among furin regulators or the involvement of alternative proteases could explain why the phenotypes observed on SARS-CoV-2 replication are modest and, absent in H5N1 infection.

In summary, we demonstrate that several previously undescribed host surface proteins have the potential to restrict SARS-CoV-2 entry, albeit with modest individual contributions. In part, this restriction can be explained by their inhibitory effect on the essential host protease furin. While we cannot exclude the possibility that these restriction factors, when acting together, may have a more potent effect. Promoting these restriction factors could present a valid target for antiviral therapies. Ongoing studies aim to further elucidate the antiviral mechanisms of the remaining proteins and their relevance in SARS-CoV-2 infection.

## Materials and methods

### Biosafety

All experiments with class 2 and 3 pathogens were performed in BSL2 or BSL3 laboratories of the University of Geneva. Experiments were approved by cantonal and federal authorities.

### Cell lines

HEK293T (human embryonic kidney, ATCC) and VeroE6 TMPRSS2 cells (provided by Dr. I. Eckerle, University of Geneva, Switzerland) were cultured in DMEM (Dulbecco’s modified eagle medium, Gibco 10566016). Calu3 (lung epithelial adenocarcinoma cells, ATCC) and Calu3 derived knockout and over expression cell lines were grown in MEM + GlutaMAX (Gibco #41090-28) + 1×MEM non-essential amino acids 100X (Gibco #11140-035) + 10 mM HEPES (Gibco #15630-056) + 1 mM sodium pyruvate (Gibco #11360-039). Cell culture media were supplemented with 10% (v/v) heat-inactivated foetal bovine serum (Gibco #10270-106. Lot: 2307592) and pen-strep antibiotics (100 U/ml penicillin and 0.1 mg/ml streptomycin, Sigma-Aldrich #P0781). For Calu3 knockout cell lines, the growth medium was supplemented with either puromycin at 4 ug/ml. All cells were maintained in low passage at 37 °C with 5% CO_2_ and 90% humidity, absence of mycoplasma was routinely confirmed by PCR.

### SDS-PAGE and western blot

For western blot analysis, the cells were lysed in protein lysis buffer (tris Hcl 1 M pH 6.8, glycerol, SDS 20%, H_2_O and DTT), sonicated 10X (30 s on and 30 s off) at 4 °C and boiled at 95 °C for 5 min. The samples were separated on a 7% SDS-PAGE gel and the transfer was performed to nitrocellulose filter membranes 0.45 uM at 120 V for 2 h. The membranes were blocked by 5% skim milk, blotted with primary antibodies followed by incubation with horseradish peroxidase HRP conjugated secondary antibodies and visualized using enhanced chemiluminescent reagent (#K12049-D50) from Advansta.

### Plasmids

pLVX-IRES-Puro was purchased from Clontech (#632183). pMD2.G and psPAX2 were a gift from Didier Trono (Addgene #12259 and #12260). pTwist-EF1α-nCoV2019-S-2xStrep and pLVX-EF1α-GFP-2xStrep-IRES-Puro were kindly gifted by Dr. Nevan Krogan’s lab UCSF, CA, USA^39^. pcDNA3.1 + /C-(K)DYK-AXL (#OHu16703D), pcDNA3.1 + /C-(K)DYK-PROM1 (#OHU19132D) and pcDNA3.1 + /C-(K)DYK-LY75 (#OHu03037) were purchased from Genscript and were cloned into pLVX-IRES-Puro for the overexpression. TMPRSS2 was kindly provided by Fabien Abdul, University of Geneva. pCAGGS-YN-GST and pCAGGS-YC-GST. Plasmids for BiFC assay for split-YFP were described previously^17^. pCAGGS Viet Nam/1203/2004 HA (with monobasic or multibasic cleavage site) was cloned based on pDZ constructs obtained from Adolfo Garcia-Sastre, Icahn School of Medicine at Mount Sinai, New York.

### Oligonucleotides for gRNA cloning and PCR primers

All oligonucleotides were purchased from Microsynth (France). Guide RNAs were designed using CRISPick and cloned into LentiCRISPR v2 (Addgene #52961) for Calu3 cells. Two gRNAs were chosen to target each gene hence the nomenclature gRNA1 and gRNA2.

**Table:** 

Target ID	Oligonucleotide sequence
NEO1 fw1	CACCGTGACACCATCAGGATTACGT
NEO1 rv1	AAACACGTAATCCTGATGGTGTCAC
NEO1 fw2	CACCGTAGGTAAGGTTGTCTCCGTG
NEO1 rv2	AAACCACGGAGACAACCTTACCTAC
AXL fw1	CACCGCTGAGAACATTAGTGCTACG
AXL rv1	AAACCGTAGCACTAATGTTCTCAGC
AXL fw2	CACCGCCTAGCAGTACATACCACCA
AXL rv2	AAACTGGTGGTATGTACTGCTAGGC
ICAM1 fw1	CACCGTGACGTGTGCAGTAATACTG
ICAM1 rv1	AAACCAGTATTACTGCACACGTCAC
ICAM1 fw2	CACCGGCCCGCTGAGGTCACGACCA
ICAM1 rv2	AAACTGGTCGTGACCTCAGCGGGCC
ADAM9 fw1	CACCGAAAAGTTTCTTATCACACGT
ADAM9 rv1	AAACACGTGTGATAAGAAACTTTTC
ADAM9 fw2	CACCGTGTGGTTTATACTTACAACA
ADAM9 rv2	AAACTGTTGTAAGTATAAACCACAC
IL1RAP fw1	CACCGGTGTCAAACCGACTATCACT
IL1RAP rv1	AAACAGTGATAGTCGGTTTGACACC
IL1RAP fw2	CACCGTCTGATGGATTCTCGCAATG
IL1RAP rv2	AAACCATTGCGAGAATCCATCAGAC
CD46 fw1	CACCGTTTGTGATCGGAATCATACA
CD46 rv1	AAACTGTATGATTCCGATCACAAAC
CD46 fw2	CACCGACTCGTAAGTCCCATTTGCA
CD46 rv2	AAACTGCAAATGGGACTTACGAGTC
LY75 fw1	CACCGGGTGGATAAGAATTAGCGAG
LY75 rv1	AAACCTCGCTAATTCTTATCCACCC
LY75 fw2	CACCGGTGGAAAGTCCAATCATGTG
LY75 rv2	AAACCACATGATTGGACTTTCCACC
PLXNA1 fw1	CACCGGTACACGAAGCCGAACATGT
PLXNA1 rv1	AAACACATGTTCGGCTTCGTGTACC
PLXNA1 fw2	CACCGGGAGAGCGAGTACATCAGTG
PLXNA1 rv2	AAACCACTGATGTACTCGCTCTCCC
PROM1 fw1	CACCGCTCACCTGCTACGACAGTCG
PROM1 rv1	AAACCGACTGTCGTAGCAGGTGAGC
PROM1 fw2	CACCGCTGTGAACCTTACACGAGCA
PROM1 rv2	AAACTGCTCGTGTAAGGTTCACAGC
ERBB2 fw1	CACCGAACTACCTTTCTACGGACGT
ERBB2 rv1	AAACACGTCCGTAGAAAGGTAGTTC
ERBB2 fw2	CACCGAGACCGTTGGACTCACGAGT
ERBB2 rv2	AAACACTCGTGAGTCCAACGGTCTC
CDH3 fw1	CACCGGTACGTTGAAGTGACCAACG
CDH3 rv1	AAACCGTTGGTCACTTCAACGTACC
CDH3 fw2	CACCGTGGTGAACATGAGGTCGTGT
CDH3 rv2	AAACACACGACCTCATGTTCACCAC
F3 fw1	CACCGCACATCCTTCACAATCTCGT
F3 rv1	AAACACGAGATTGTGAAGGATGTGC
F3 fw2	CACCGCAGGAGCGTCCGAGCGACGG
F3 rv2	AAACCCGTCGCTCGGACGCTCCTGC
MYOF fw1	CACCGACCACACTCATAGACGGCGA
MYOF rv1	AAACTCGCCGTCTATGAGTGTGGTC
MYOF fw2	CACCGGGAGTACAAGACCTGATGTG
MYOF rv2	AAACCACATCAGGTCTTGTACTCCC
SDK1 fw1	CACCGGAGCACGTCTAGGATCGTGG
SDK1 rv1	AAACCCACGATCCTAGACGTGCTCC
SDK1 fw2	CACCGGTTTCATGGATACGGACCAG
SDK1 rv2	AAACCTGGTCCGTATCCATGAAACC

### Antibodies

Mouse monoclonal against actin antibody (#ab49900), rabbit monoclonal against ACE2 antibody (#ab272500, epr24705-45), rabbit monoclonal against ERBB2 antibody (#ab134182) were purchased from Abcam. The streptavidin-HRP antibody (#S-911) were purchased from Thermofisher. Mouse monoclonal anti-FLAG HRP antibody (#A8592), goat polyclonal anti-rabbit IgG HRP antibody (#A8275) and goat polyclonal anti-mouse IgG HRP antibody (#A5278) were purchased from Sigma-Aldrich. Rabbit monoclonal against ICAM1 (#ab282575), rabbit recombinant multiclonal against CD133/PROM1 antibody (#ab278053), rabbit monoclonal against AXL antibody (#ab259831) and rabbit monoclonal against LY75 antibody (#ab124897) were purchased from Abcam. Mouse monoclonal against Spike S2 antibody (#GTX632604**)** was purchased from Genetex.

### Recombinant proteins

Trimeric Spike was previously described^40^. Based on this sequence (GenBank accession no. MN908947.3 for the original sequence), we added the coding sequence for HRP on the 3′ end of the T4foldon, connected via a GSGSG-linker and followed by a His10-tag. A trimerized HRP control was designed with the same T4foldon and His10 tag. The recombinant proteins were expressed and purified at the Protein core facility (CMU, University of Geneva) using the baculovirus (Sf9 insect cells) expression system. Baculovirus were generated using a modified pFastBac vector encoding C-terminally tagged (i) Wuhan Spike, (ii) no protein. The proteins had a C-terminal tag composed of fused Wuhan Spike—HRP and a 10-histidine tag. The media containing the proteins was centrifuged at 4000×*g* for 15 min at 4 °C and filtered using 0.22 µm filters. The media was concentrated and adjusted to 10 mM imidazole and applied on to a 5 ml His-trap FF column (Cytiva). 100 ml of PBS supplemented with 1M NaCl and 10 mM Imidazole was used to wash the column and the column was eluted with 15 ml of elution buffer (1×PBS, 200 mM NaCl, 450 mM imidazole). Proteins eluted were concentrated to 1 ml using AMICON 30 MWCO concentrators and loaded on a Size Exclusion Chromatography Superdex 200 10/300 column equilibrated in PBS at 4 °C. Pure protein fractions were pooled, concentrated and flash frozen in liquid nitrogen.

### Cell surface proximity ligation assay

Calu3 cells were grown in 6-well format and incubated with 100 µg of recombinant Spike-HRP or HRP alone for 60 min. Biotin phenol and H_2_O_2_ were added for 10 min to allow proximity ligation of biotin. Cells were quenched and lysed with lysis buffer (0.4% SDS, 500 mM NaCl, 5 mM EDTA, 50 mM Tris–HCl pH 7.5, 1% Triton-X100, 1 mM DTT, protease inhibitor). Biotinylated proteins were precipitated with streptavidin-agarose beads (Thermofisher #11205D) and prepared for mass spectrometry by on bead trypsin digest.

### Mass spectrometry

On bead trypsin digestion was done to digest the proteins and peptides were analysed by nanoLC-MSMS using an easynLC1000 (Thermo Fisher) coupled to a Qexactive Plus mass spectrometer (Thermo Fisher). Data were analysed with Scaffold (Proteome Software) with 1% of protein FDR with a 0.1% of peptide FDR. The initial hits obtained from two independent experiments in Calu3 cells were filtered using the cell surface proteome atlas^41^, narrowing down potential hits to highly probable surface proteins. Contaminants from precipitation experiments were identified with the CRAPome database^42^. Only proteins present in five or fewer of 716 biotinylation/pulldown datasets were considered as valid hits. We further refined the list to proteins that were identified with at least five peptides and showed a 2.5-fold enrichment in SARS-CoV-2-S-HRP samples compared to HRP controls.

### Generation of a KO cell line using CRISPR/Cas9 with lentiviral transduction for Calu3

Subconfluent HEK293T cells in 6-well plates were transfected at a ratio of 1:3:4 with the following plasmids pMD2.G (vesicular stomatitis virus G protein (VSV G)), psPAX2 (HIV gag-pol) were a gift from Didier Trono (Addgene plasmid # 12259 and #12260) and lentiCRISPRv2, a gift from Feng Zhang (Addgene plasmid # 52961)^9^ containing the specific gRNA (see above) with 2 µg/µl of Trans-lT LT1 (Mirus). After 24 h, HEK293T medium was replaced with target cell medium. Calu3 cells were seeded in 6-well plates at a density of 50%. The HEK293T supernatants containing lentiviruses to knockout the target protein was harvested 48 h post transfection with a syringe and was passed through a sterile filter of 0.45 um and complemented with polybrene at 8 µg/ml. The supernatant-polybrene mix was added to Calu3 cells after washing the cells 1X with PBS. The supernatant containing lentiviruses was removed from Calu3 cells after 4 h and replaced with respective growth medium. At 48 h post transduction, Calu3 cells were split and selected with puromycin at 4 µg/ml. The knockout efficiency was assessed using western blot.

### SARS-CoV-2 virus-like particles (VLP) production

To generate replication incompetent, luciferase expressing VLP, subconfluent 100 mm dish 293T cells were transfected with: 10 µg of psPAX, 5 µg of pCG1 SARS-CoV-2 Spike^7^ (mono or multibasic variant) or 2.5 µg of pMD2.G and 15 µg of CD510B Gluc (vector backbone pCDH-CMV-MCS-EF1-Puro from Sanbio, Netherlands #CD510B-1) kindly provided by Fabien Abdul, University of Geneva, using Trans lT-LT1 transfection reagent (Mirus) according to manufacturer’s instructions. The supernatant containing SARS-CoV-2 Spike and VSV G pseudotyped VLP were harvested 48 h and 72 h post transfection, respectively. The supernatants were cleared from cell debris by centrifugation (2000×*g*, 10 min, 4 °C) and were passed through a 0.45 µm filter attached to a syringe to remove cell debris. Aliquots were stored at − 80 °C. VLP stocks were titered on target cells to achieve comparable infection rates within the linear range of the luciferase assay.

### Titration of SARS-CoV-2 and VSV G VLPs on Cas9 empty and ACE2KO cells

Subconfluent Calu3 Cas9 empty, Calu3 ACE2KO and Calu3 ACE2*KO in 96-well plates were transduced with different volumes of the both SARS-CoV-2 and VSV G VLPs. 6 h post transfection, the VLPs were removed from the cells, washed 2X with 300 ul of PBS per well and replaced with appropriate growth medium. The supernatant was collected every 24 h until 96 h, washed 2X with PBS and replaced with growth medium. After the harvest, the supernatants were centrifuged at 1500×*g* for 7 min. 5 µl of the supernatant was then mixed with 50 µl of coelenterazine (Biosynth #EC175526) on white plates and the luciferase activity was measured using the Dual Glo protocol on the Glomax 96-well microplate luminometer.

### Infection with SARS-CoV-2 VLP

Calu3 cells were seeded to achieve subconfluency in 96-well plates pre-coated with poly-L-lysine. The cells were washed once with PBS and infected with 100 µl of SARS-CoV-2 Spike or VSV G pseudotyped VLP per well. 6 h post-infection, the VLP were removed, the cells were washed twice with PBS and 100 ul of fresh medium (MEM + GlutaMAX (Gibco #41090-28) + 1×MEM non-essential amino acids 100X (Gibco #11140-035) + 10 mM HEPES (Gibco #315630-056) + 1 mM sodium pyruvate (Gibco #11360-039) + 10% (v/v) heat-inactivated foetal bovine serum (Gibco #10270-106. Lot: 2307592) + pen-strep antibiotics (100 U/ml penicillin and 0.1 mg/ml streptomycin, Sigma-Aldrich #P0781)) was added to each well. 96 h post-infection, the supernatants were collected and subjected to centrifugation. The gaussia luciferase activity was measured by adding 5 µl of the supernatant with 50 µl of coelenterazine (Biosynth #EC175526) on white plates using the Glomax 96-well microplate luminometer Promega. DualGlo protocol in the Glomax software was used for the measurement which was at a rate of 1 s per well.

### SARS-CoV-2 virus production

Calu3 cells were grown in 100 mm dish at sub-confluency. The cells were infected with an MOI of 0.01 of Omicron and Wuhan (kindly provided by Prof. Isabella Eckerle) viruses for 1 h at 37 °C in MEM + GlutaMAX (Gibco #41090-28) + 1×MEM non-essential amino acids 100X (Gibco #11140-035) + 10mM HEPES (Gibco #15630-056) + 1 mM sodium pyruvate (Gibco #311360-039) + pen-strep antibiotics (100 U/ml penicillin and 0.1 mg/ml streptomycin, Sigma-Aldrich #P0781) + 2% (v/v) heat-inactivated fetal bovine serum (Gibco #10270-106. Lot: 2307592). The inoculum was removed after 1 h and fresh medium was added. The viral supernatants were recovered 48 h and 96 h post-infection for Omicron and Wuhan, respectively. The supernatants were centrifuged at 450×*g* for 5 min and stored 

at − 70 °C.

### Plaque assay to determine viral titer of SARS CoV 2

Vero E6 TMPRSS2 cells were grown to form a monolayer in 24-well plates. The cells were infected with 200 µl of serially diluted viruses or supernatants collected post viral infections. Virus or the supernatant were diluted in serum free DMEM medium (Gibco #10566016). 1 h post infection, the inoculum was removed and 2.4% Avicel overlay (Dupont) was added to the cells. Cells were incubated for 96h for both viruses at 37 °C. The overlay was removed, then cells were fixed in 4% formaldehyde and the cell monolayer was stained with a solution of crystal violet. Plaques were counted and multiplied with the dilution and volume factor to determine viral titers (pfu/ml).

### Bla-M1 IAV VLP fusion assay

Bla-M1 VLP fusion assays were performed as described^43^ on Calu3 cells. Plasmids were kindly provided by Adolfo Garcia Sastre, Icahn School of Medicine at Mount Sinai, New York, NY. VLP were not activated by TPCK trypsin, since Calu3 cells express furin and TMPRSS2.

### Rescue of recombinant IAV

Influenza A virus was rescued using plasmid based reverse genetics^44^. Plasmids to rescue low pathogenic or high pathogenic A/Viet Nam/1203/2004 were previously described^45^ and kindly provided by Adolfo Garcia Sastre, Icahn School of Medicine at Mount Sinai, New York, NY. Rescued virus stocks were sequenced. IAV stocks were grown and titered on MDCK cells^44^.

### Co-Immunoprecipitation assay

Subconfluent 293T cells were transfected in 6 well plates using Trans lT-LT1 transfection reagent (Mirus) according to manufacturer’s instructions. 24 h post transfection, cells were lysed using IP lysis buffer (50 mM Tris pH = 7.5, 150 mM NaCl, 5mM EDTA, 0.5% NP-40 and H_2_O) for 20 min on ice and sonicated 10X (30 s on and 30 s off) at 4 °C. The supernatant was collected after centrifugation at 10,000×*g* for 20 min at 4 °C. 10% of the supernatant was used to verify the input proteins by western blot. For the pull down, the supernatants were diluted in IP lysis buffer in a ratio of 1:6 and incubated with streptavidin-agarose beads (Thermofisher #11205D) overnight at 4 °C under agitation. 24h post incubation, the beads were washed 3X with IP lysis buffer and boiled with protein lysis buffer at 95 °C for 2 min. Finally, the supernatant was stored at − 20 °C for western blot analysis.

### Bimolecular fluorescence complementation (BiFC) based cell fusion assay

Sub-confluent Vero E6 cells were transfected in 6 well plates at a ratio of 1:1 with the following plasmids according to (Table 1) using Trans lT-LT1 transfection reagent (Mirus) according to manufacturer’s instructions. 24 h post-transfection, cells were detached with trypsin–EDTA and re-seeded in 12 wells at a ratio of 1:1 in indicated combinations: 1 + 3, 1 + 2, 1 + 4, 1 + 5, 3 + 2, 3 + 6, 3 + 7, 6 + 2, 7 + 2, 4 + 2 and 5 + 2. 72 h post re-seeding, the cells were washed 1X with PBS and fixed with 4% formaldehyde for 20 min. The fixed cells were then washed 2X with PBS and permeabilized using PBS 1% Triton (Thermofisher # 215680010) for 10 min. Then the cells were washed 2X with PBS and stained with DAPI for 20 min in the dark at RT. After staining, cells were then washed 2X with PBS and 1ml of PBS was added to each well. The plates were stored in dark until imaging using ImageExpress plate reader (Molecular Devices). The images were analyzed using the software MetaXpress Version 6.6.1.42.

**Table 1:** List of plasmid combinations used for the BiFC assay.

Combination number	Plasmid 1	Plasmid 2	Plasmid 3
1	pTwist-EF1α-nCoV2019-S-2xStrep	pCAGGS-YN-GST	
2	pLVX.IRES-Puro	pCAGGS-YC-GST	
3	CD509B-TMPRSS2	pCAGGS-YC-GST	
4	CD509B-TMPRSS2	pCAGGS-YC-GST	pLVX-IRES-Puro-PROM1
5	CD509B-TMPRSS2	pCAGGS-YC-GST	pLVX-IRES-Puro-AXL
6	pTwist-EF1α-nCoV2019-S-2xStrep	pCAGGS-YN-GST	pLVX-IRES-Puro-PROM1
7	pTwist-EF1α-nCoV2019-S-2xStrep	pCAGGS-YN-GST	pLVX-IRES-Puro-AXL

### SARS-CoV-2 infection

Calu3 knockout (ACE2, PROM1 and AXL) were seeded in 24 well plates to achieve subconfluency. They were infected with Omicron (BA.1: EPI_ISL_7605546) SARS-CoV-2 virus at a MOI of 0.1. After 45 min of incubation with the virus, the inoculum was removed, the cells were washed 1X with PBS. The wells were replaced by respective growth medium with 2% FBS + 1% P/S. 72 h post infection, the supernatants were collected for plaque assays to determine the viral titer.

### Statistics

Statistical analysis was performed using GraphPad Prism 9. Statistical tests applied are indicated in each respective figure legend.

## Supplementary Information

Supplementary Information 1.

Supplementary Information 2.

Supplementary Information 3.

Supplementary Information 4.

Supplementary Information 5.

Supplementary Information 6.

Supplementary Information 7.

Supplementary Information 8.

## Data Availability

The datasets used and/or analysed during the current study available from the corresponding author on reasonable request.
