# Supplementary material for: Identification of host cell surface proteins inhibiting furin dependent proteolytic processing of viral glycoproteins
Source: Sci Rep. 2025 Jul 15;15:25454. doi: 10.1038/s41598-025-11164-x (PMC12259872; doi:10.1038/s41598-025-11164-x)
Supplement: Supplementary file 1 — Supplementary Information 1. [file 41598_2025_11164_MOESM1_ESM.pdf]

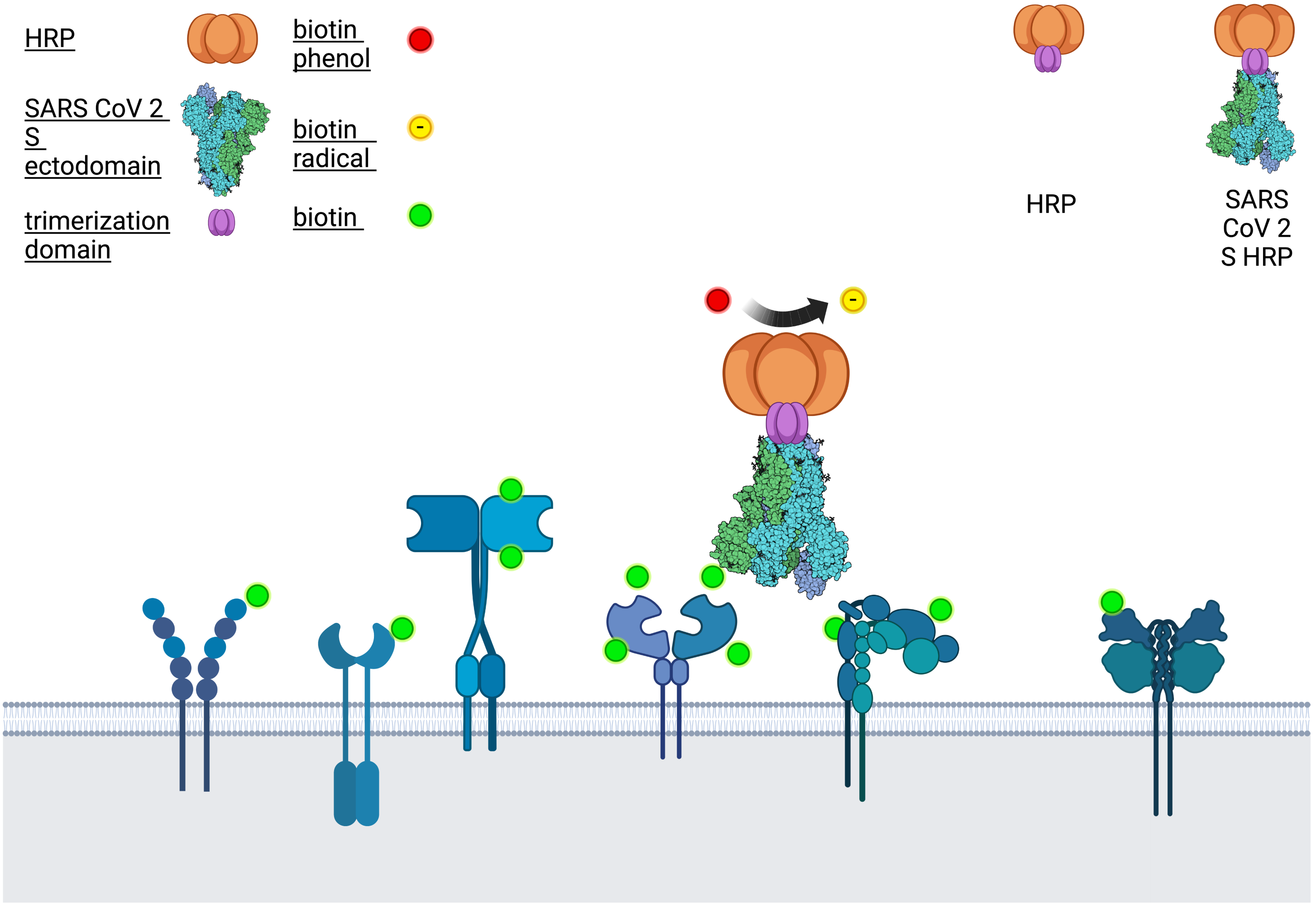
HRP
SARS CoV 2 S ectodomain
trimerization domain
biotin phenol
biotin radical
biotin
HRP
SARS CoV 2 S HRP
