# Supplementary material for: Identification of host cell surface proteins inhibiting furin dependent proteolytic processing of viral glycoproteins
Source: Sci Rep. 2025 Jul 15;15:25454. doi: 10.1038/s41598-025-11164-x (PMC12259872; doi:10.1038/s41598-025-11164-x)
Supplement: Supplementary file 2 — Supplementary Information 2. [file 41598_2025_11164_MOESM2_ESM.pdf]

A

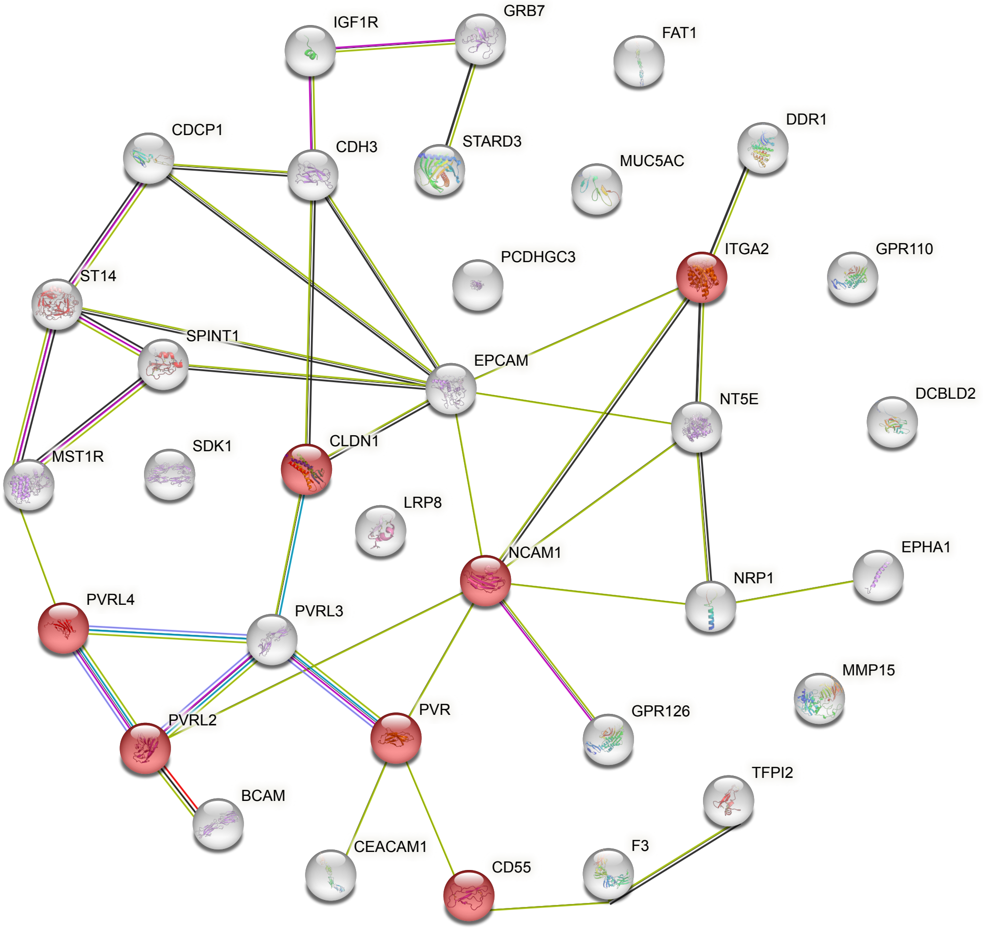

B

Biological Process				
GO term	description	count	strength	FDR
GO:0022610	Biological adhesion	22 of 931	1.11	2.59E-16
GO:0007155	Cell adhesion	21 of 925	1.09	3.28E-15
GO:0044409	Entry into host	12 of 103	1.8	4.39E-15
GO:0040011	Locomotion	22 of 1251	0.98	2.56E-14
GO:0031589	Cell-substrate adhesion	11 of 182	1.52	4.93E-11

Molecular Function				
GO term	description	count	strength	FDR
GO:0050839	Cell adhesion molecule binding	17 of 538	1.23	6.41E-14
GO:0001618	Virus receptor activity	10 of 74	1.87	4.51E-13
GO:0005178	Integrin binding	9 of 147	1.52	6.44E-09
GO:0038023	Signaling receptor activity	18 of 1453	0.83	8.20E-09
GO:0005102	Signaling receptor binding	16 of 1581	0.74	2.46E-06
