# Supplementary figures and images for: Identification of host cell surface proteins inhibiting furin dependent proteolytic processing of viral glycoproteins
Source: Sci Rep. 2025 Jul 15;15:25454. doi: 10.1038/s41598-025-11164-x (PMC12259872; doi:10.1038/s41598-025-11164-x)

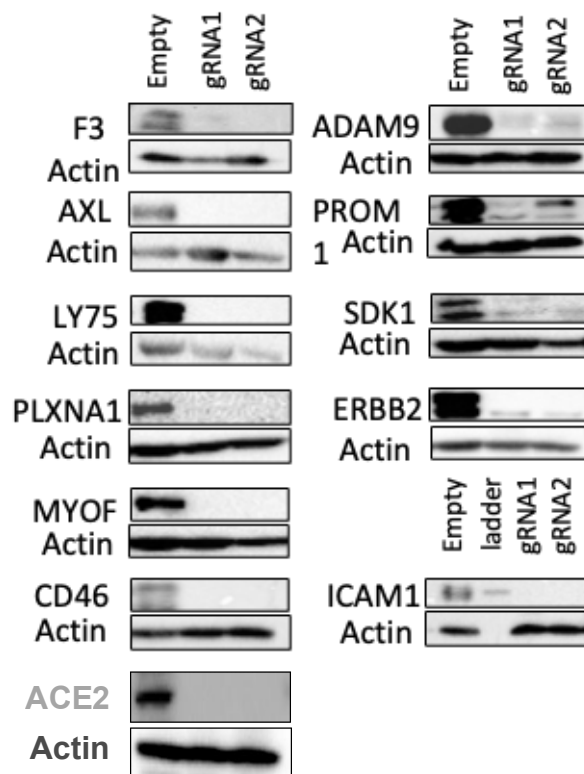

Supplement: Supplementary file 3 — Supplementary Information 3. [file 41598_2025_11164_MOESM3_ESM.pdf]

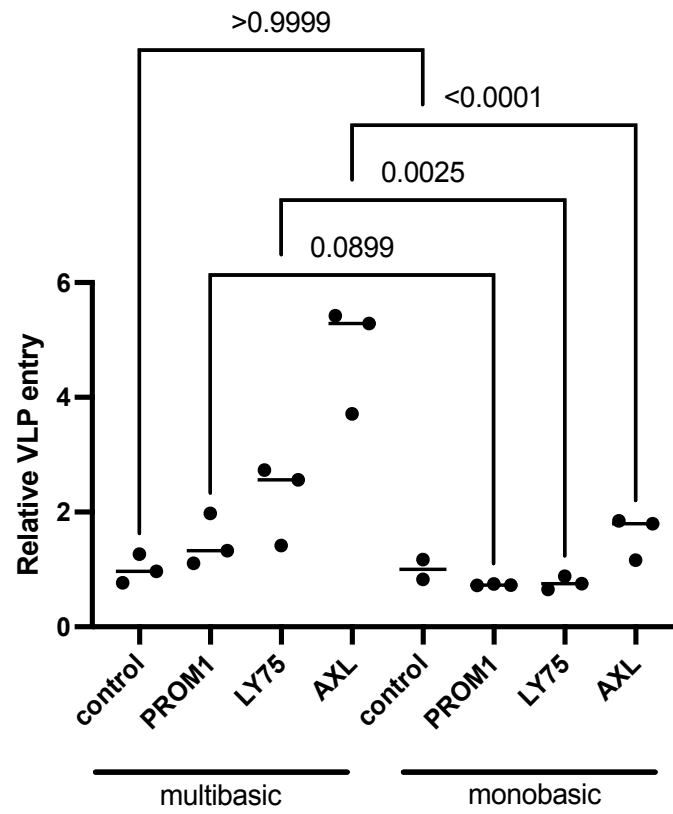

Supplement: Supplementary file 4 — Supplementary Information 4. [file 41598_2025_11164_MOESM4_ESM.pdf]

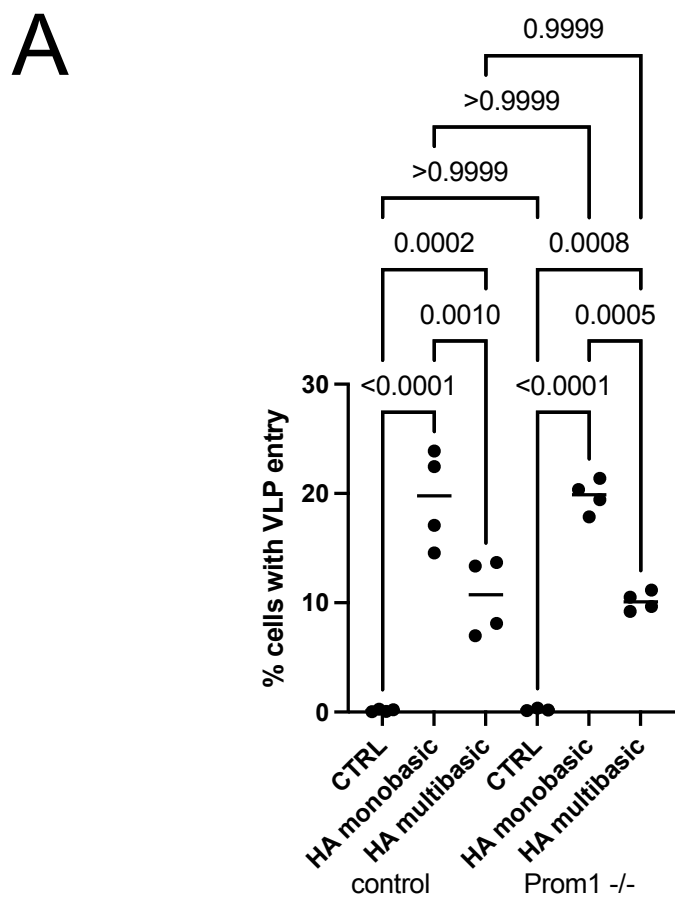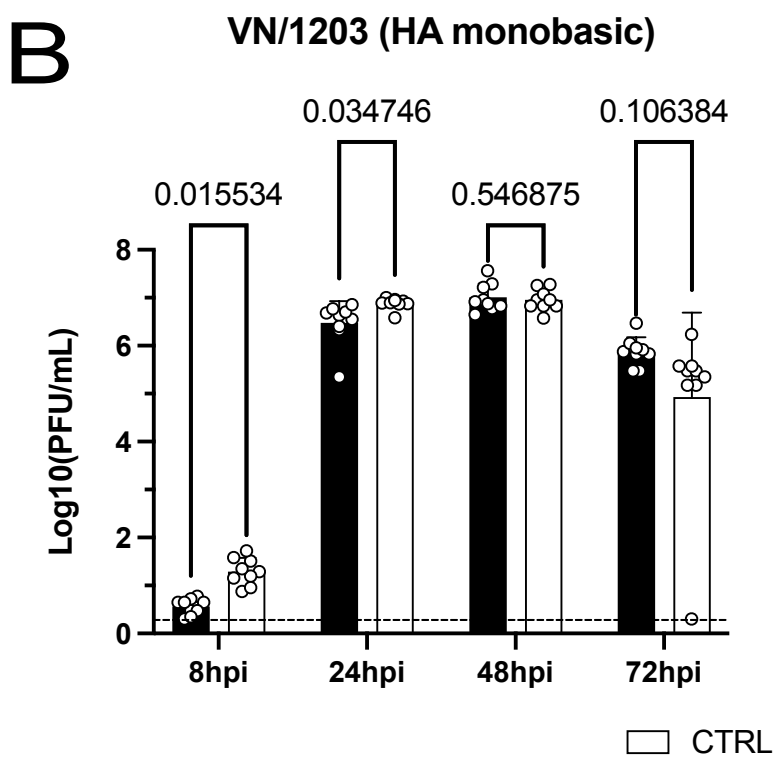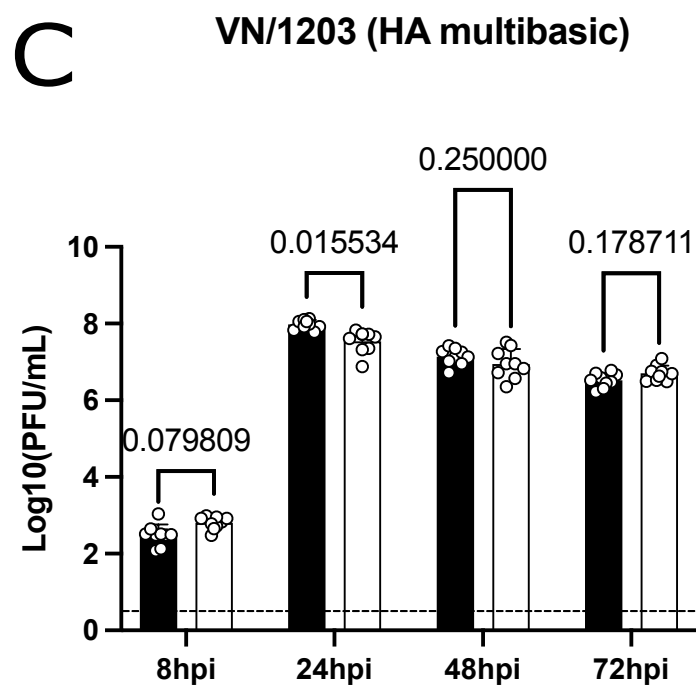

Supplement: Supplementary file 5 — Supplementary Information 5. [file 41598_2025_11164_MOESM5_ESM.pdf]
