# Supplementary material for: Identification of host cell surface proteins inhibiting furin dependent proteolytic processing of viral glycoproteins
Source: Sci Rep. 2025 Jul 15;15:25454. doi: 10.1038/s41598-025-11164-x (PMC12259872; doi:10.1038/s41598-025-11164-x)
Supplement: Supplementary file 6 — Supplementary Information 6. [file 41598_2025_11164_MOESM6_ESM.pdf]

Supplementary Figure legends

Figure S1: Graphical summary of CSPL technique generated with BioRender.com.

Figure S2: A) String network of the 34 proteins found only in individual mass spectrometry experiments (defined as background). **B)** Gene ontology classification of the proteins mentioned in (A) and sorted from lowest to highest false discovery rate (FDR). Count refers to the number of proteins annotated in a particular network over the total number of proteins in this network. Strength (indicator of enrichment) indicates the ratio of proteins from the network assigned to a term over the expected number of proteins assigned to a random network of equal size.

Figure S3: Total cell lysates were separated by SDS-PAGE and analyzed by western blot to confirm knockouts of target proteins in Calu3 cells. ACE2 knockout in Calu3 cells was done as a positive control. Equal loading was verified by probing for beta-actin. The different gRNAs are mentioned as gRNA1 and gRNA2.

Figure S4: SARS-CoV-2 pseudotyped VLP entry (mono and multibasic cleavage site) into Calu3 KO cells. A) SARS-CoV-2 pseudotyped VLP entry in Calu3 knockout cells using Spike with monobasic or multibasic cleavage site The results are shown in fold change to control in relative light units. P-values were calculated with One way ANOVA.

Figure S5: A) IAV-VLP entry assay. Control and PROM1 deficient Calu3 cells were infected with A/WSN/1933 based VLP pseudotyped with A/Viet Nam/1203/2004 hemagglutinin (monobasic or multibasic version). Percentage of positive cells is indicated from two

independent experiments with two biological replicates each. Statistical significance was determined by multiple one-way ANOVA testing. *P*-values for indicated sample pairs are provided. **B** and **C)** Control and PROM1 deficient Calu3 were infected for indicated timepoints with 0.01 MOI of A/Viet Nam/1203/2004 (low pathogenic variant with monobasic cleavage site in the HA) (**B**), or 0.01 MOI of A/Viet Nam/1203/2004 (high pathogenic variant with multibasic cleavage site in the HA). Data from three independent viral growth curves with two biological replicates each are indicated. Statistical significance was determined by multiple one-way ANOVA testing. *P*-values for indicated sample pairs are provided.
