# Supplementary material for: Identification of host cell surface proteins inhibiting furin dependent proteolytic processing of viral glycoproteins
Source: Sci Rep. 2025 Jul 15;15:25454. doi: 10.1038/s41598-025-11164-x (PMC12259872; doi:10.1038/s41598-025-11164-x)
Supplement: Supplementary file 7 — Supplementary Information 7. [file 41598_2025_11164_MOESM7_ESM.pdf]

TABLE S1: Filtering steps applied to raw data from CSPL experiment after exclusion of non surface proteins.

1st filtering

1st experiment (SARS-CoV-2 HRP 5 peptides or more list)

Alternate.ID	Calu3.HRP	Calu3.SARSCOV2.HRP	ratio
PLEC	30	300	10
ACACA	2	144	72
KRT8	12	95	7.916666667
DSP	25	94	3.76
PLXNB2	16	84	5.25
ERBB2	26	83	3.192307692
KRT19	24	81	3.375
KRT18	12	70	5.833333333
PC	0	67	#DIV/0!
PRKDC	0	66	#DIV/0!
ITGB1	17	63	3.705882353
MUC5AC	2	61	30.5
ITGB4	8	48	6
MCCC1	0	47	#DIV/0!
TFRC	13	46	3.538461538
ITGA6	10	43	4.3
EPHA2	11	42	3.818181818
CDH1	9	39	4.333333333
KRT7	6	38	6.333333333
MET	4	35	8.75
ITGAV	9	35	3.888888889
ALCAM	10	35	3.5
CD44	13	35	2.692307692
DSG2	2	33	16.5
ITGA3	9	33	3.666666667
KRT13	0	30	#DIV/0!
FASN	0	28	#DIV/0!
TUBB	5	27	5.4
PTPRK	0	25	#DIV/0!
UBB	4	25	6.25
PCCA	0	24	#DIV/0!
TUBA3C	0	24	#DIV/0!
TUBB4B	0	24	#DIV/0!
TUBA1B	7	24	3.428571429
PTPRB	0	23	#DIV/0!
PTPRF	0	22	#DIV/0!
MKI67	0	22	#DIV/0!
ADAM10	4	22	5.5
PTK7	0	21	#DIV/0!
EPPK1	0	20	#DIV/0!
CCT5	2	20	10
TCP1	3	20	6.666666667
TUBB4A	0	19	#DIV/0!
ITGB5	0	19	#DIV/0!
CEMIP2	0	19	#DIV/0!

ATP1A1	2	19	9.5
HSPA8	6	19	3.166666667
ADGRL2	2	18	9
CCT8	4	18	4.5
CCT4	4	18	4.5
HSPA1A	5	17	3.4
PROM1	0	16	#DIV/0!
FLNA	0	16	#DIV/0!
CCT3	0	16	#DIV/0!
PLXNA1	0	16	#DIV/0!
PKM	6	16	2.666666667
GLG1	0	15	#DIV/0!
CD46	2	15	7.5
CLTC	2	15	7.5
EGFR	0	14	#DIV/0!
HSP90AA1	0	14	#DIV/0!
KRT4	0	14	#DIV/0!
SLC3A2	3	14	4.666666667
HIST1H4D	5	14	2.8
CCT2	5	14	2.8
KIF23	0	13	#DIV/0!
IGF1R	0	13	#DIV/0!
ROR1	0	13	#DIV/0!
AHNAK	0	13	#DIV/0!
CCT7	2	13	6.5
LY75	0	12	#DIV/0!
TOP2A	0	12	#DIV/0!
ATP2A2	0	12	#DIV/0!
ACE2	0	12	#DIV/0!
IL1RAP	0	12	#DIV/0!
BANF1	2	12	6
ITGB6	2	12	6
CCN1	0	11	#DIV/0!
OCLN	0	11	#DIV/0!
POF1B	0	11	#DIV/0!
KRT23	0	11	#DIV/0!
ATP1B1	3	11	3.666666667
LSR	3	11	3.666666667
CCT6A	4	11	2.75
EEF1A2	0	10	#DIV/0!
SDK1	0	10	#DIV/0!
DCBLD2	0	10	#DIV/0!
GTPBP4	0	10	#DIV/0!
RPS3	3	10	3.333333333
LGALS3	4	10	2.5
ICAM1	0	9	#DIV/0!
HNRNPA1	0	9	#DIV/0!
MMP15	0	9	#DIV/0!

RACK1	0	9	#DIV/0!
HSPG2	0	9	#DIV/0!
F11R	0	9	#DIV/0!
DDX3X	0	8	#DIV/0!
SUN1	0	8	#DIV/0!
ARF4	0	8	#DIV/0!
MYH9	0	8	#DIV/0!
TUFM	0	8	#DIV/0!
RPL6	0	8	#DIV/0!
PCDH1	0	8	#DIV/0!
LBR	0	8	#DIV/0!
NEO1	0	8	#DIV/0!
RACGAP1	0	8	#DIV/0!
DPP4	2	8	4
F3	0	7	#DIV/0!
CDH3	0	7	#DIV/0!
CAD	0	7	#DIV/0!
AXL	0	7	#DIV/0!
IQGAP1	0	7	#DIV/0!
ANXA11	0	7	#DIV/0!
ARF3	0	7	#DIV/0!
DHX9	0	7	#DIV/0!
DDR1	0	7	#DIV/0!
ADAM9	0	7	#DIV/0!
TSPAN14	0	7	#DIV/0!
SYNE2	0	7	#DIV/0!
MYADM	0	7	#DIV/0!
NAT10	0	7	#DIV/0!
MYOF	0	7	#DIV/0!
ROBO1	0	7	#DIV/0!
EZR	2	7	3.5
PRDX6	2	7	3.5
RPS9	2	7	3.5
RAB7A	2	7	3.5
GOLIM4	0	6	#DIV/0!
NRP1	0	6	#DIV/0!
CTNND1	0	6	#DIV/0!
CLDN1	0	6	#DIV/0!
EPRS	0	6	#DIV/0!
NCAM1	0	6	#DIV/0!
PVR	0	6	#DIV/0!
ABCD3	0	6	#DIV/0!
RPL3	0	6	#DIV/0!
SLC12A2	0	6	#DIV/0!
JAG1	0	6	#DIV/0!
STARD3	0	6	#DIV/0!
NECTIN2	0	6	#DIV/0!
RPL7	2	6	3

SYNCRIP	0	5	#DIV/0!
LDLR	0	5	#DIV/0!
RPN1	0	5	#DIV/0!
SLC25A5	0	5	#DIV/0!
LDHB	0	5	#DIV/0!
SLC25A6	0	5	#DIV/0!
HIST1H1B	0	5	#DIV/0!
RPL9P8	0	5	#DIV/0!
RAB5C	0	5	#DIV/0!
ITGA1	0	5	#DIV/0!
RAB1A	0	5	#DIV/0!
YBX1	0	5	#DIV/0!
DSC2	0	5	#DIV/0!
PTPRJ	0	5	#DIV/0!
SQSTM1	0	5	#DIV/0!
DAG1	0	5	#DIV/0!
DYNC1H1	0	5	#DIV/0!
FAT1	0	5	#DIV/0!
ADGRG6	0	5	#DIV/0!
NECTIN4	0	5	#DIV/0!
CDCP1	0	5	#DIV/0!
NECTIN3	0	5	#DIV/0!
DDX21	0	5	#DIV/0!
PCDHGC3	0	5	#DIV/0!

2nd experiment (SARS-CoV-2 HRP 5 peptides or more)

Alternate.ID	Calu3.HRP	Calu3.SARSCOV2.HRP	ratio 2
ERBB2	0	74	#DIV/0!
ITGB4	0	60	#DIV/0!
ACACA	0	52	#DIV/0!
PLXNB2	0	51	#DIV/0!
ITGB1	0	50	#DIV/0!
ITGA6	0	45	#DIV/0!
TFRC	0	36	#DIV/0!
PLEC	0	31	#DIV/0!
ITGA2	0	29	#DIV/0!
PC	0	29	#DIV/0!
KRT5	0	29	#DIV/0!
ITGAV	0	28	#DIV/0!
EPHA2	0	28	#DIV/0!
MET	0	27	#DIV/0!
ADAM10	0	26	#DIV/0!
ITGA3	0	24	#DIV/0!
ACTB	8	24	3
PTPRF	0	23	#DIV/0!
CDH1	0	23	#DIV/0!
CD44	0	23	#DIV/0!
MCCC1	0	23	#DIV/0!
ATP1A1	0	21	#DIV/0!
KRT6A	0	21	#DIV/0!
UBB	0	21	#DIV/0!
ALCAM	0	19	#DIV/0!
SLC3A2	0	17	#DIV/0!
PROM1	0	16	#DIV/0!
DSG2	0	16	#DIV/0!
PTPRK	0	16	#DIV/0!
HSP90AB1	0	16	#DIV/0!
KRT14	0	16	#DIV/0!
JUP	0	16	#DIV/0!
PKM	5	16	3.2
KRT19	0	15	#DIV/0!
DSP	0	15	#DIV/0!
FASN	0	15	#DIV/0!
AHNAK	0	15	#DIV/0!
KRT16	0	14	#DIV/0!
TUBA3C	0	14	#DIV/0!
EGFR	0	13	#DIV/0!
ATP1B1	0	13	#DIV/0!
PTPRJ	0	13	#DIV/0!
PLXNA1	0	13	#DIV/0!
CCT4	0	13	#DIV/0!
TUBA1A	0	13	#DIV/0!

LY75	0	12	#DIV/0!
TUBB	0	12	#DIV/0!
CCN1	0	12	#DIV/0!
ITGB6	0	11	#DIV/0!
PTPRB	0	11	#DIV/0!
PTK7	0	11	#DIV/0!
MYH9	0	11	#DIV/0!
CLTC	0	11	#DIV/0!
PCCA	0	11	#DIV/0!
ANXA2	0	11	#DIV/0!
TCP1	0	11	#DIV/0!
EEF1A1	4	11	2.75
TFPI2	0	10	#DIV/0!
CCT8	0	10	#DIV/0!
CCT2	0	10	#DIV/0!
OCLN	0	10	#DIV/0!
KRT77	0	10	#DIV/0!
HLA-A	0	9	#DIV/0!
CD59	0	9	#DIV/0!
CD46	0	9	#DIV/0!
DPP4	0	9	#DIV/0!
ADAM9	0	9	#DIV/0!
IL1RAP	0	9	#DIV/0!
KRT8	0	9	#DIV/0!
HSPA1A	0	9	#DIV/0!
RPL6	0	9	#DIV/0!
SRSF1	0	9	#DIV/0!
PRPF4B	0	9	#DIV/0!
SRRM2	0	9	#DIV/0!
ADGRL2	0	8	#DIV/0!
CEACAM1	0	8	#DIV/0!
EPCAM	0	8	#DIV/0!
ITGB5	0	8	#DIV/0!
MYOF	0	8	#DIV/0!
CEMIP2	0	8	#DIV/0!
TOP1	0	8	#DIV/0!
PRKDC	0	8	#DIV/0!
LSR	0	8	#DIV/0!
CCT7	0	8	#DIV/0!
HSP90AA1	2	8	4
KRT18	3	8	2.666666667
LDLR	0	7	#DIV/0!
RPN1	0	7	#DIV/0!
NT5E	0	7	#DIV/0!
AXL	0	7	#DIV/0!
MST1R	0	7	#DIV/0!
ENSG000002627	0	7	#DIV/0!
PRPF40A	0	7	#DIV/0!

LUC7L3	0	7	#DIV/0!
P4HB	0	7	#DIV/0!
KRT7	0	7	#DIV/0!
EEF2	0	7	#DIV/0!
CCT5	0	7	#DIV/0!
RACK1	0	7	#DIV/0!
SRSF11	0	7	#DIV/0!
PRDX1	0	7	#DIV/0!
KPRP	0	7	#DIV/0!
DDX46	0	7	#DIV/0!
ICAM1	0	6	#DIV/0!
EPHB2	0	6	#DIV/0!
ITGA1	0	6	#DIV/0!
JAG1	0	6	#DIV/0!
ROR1	0	6	#DIV/0!
SRSF3	0	6	#DIV/0!
FLG2	0	6	#DIV/0!
HIST1H2BK	0	6	#DIV/0!
PGK1	0	6	#DIV/0!
EPRS	0	6	#DIV/0!
ANXA4	0	6	#DIV/0!
CCT3	0	6	#DIV/0!
SPTAN1	0	6	#DIV/0!
LRP8	0	6	#DIV/0!
SRRM1	0	6	#DIV/0!
MYADM	0	6	#DIV/0!
CP	0	5	#DIV/0!
CD55	0	5	#DIV/0!
EPHA1	0	5	#DIV/0!
CD97	0	5	#DIV/0!
BCAM	0	5	#DIV/0!
EPHB4	0	5	#DIV/0!
HNRNPK	0	5	#DIV/0!
PCDH1	0	5	#DIV/0!
LMAN2	0	5	#DIV/0!
DAG1	0	5	#DIV/0!
ADGRF1	0	5	#DIV/0!
NEO1	0	5	#DIV/0!
KIRREL1	0	5	#DIV/0!
F11R	0	5	#DIV/0!
TSPAN14	0	5	#DIV/0!
ST14	0	5	#DIV/0!
HSP90B1	0	5	#DIV/0!
U2SURP	0	5	#DIV/0!
SPINT1	0	5	#DIV/0!
CTNND1	0	5	#DIV/0!
LDHA	0	5	#DIV/0!
ANXA1	0	5	#DIV/0!

HIST1H2AI	0	5	#DIV/0!
HIST1H1E	0	5	#DIV/0!
ATP2A2	0	5	#DIV/0!
RPS3	0	5	#DIV/0!
HNRNPH1	0	5	#DIV/0!
PHB	0	5	#DIV/0!
HSPA9	0	5	#DIV/0!
ALDH1A3	0	5	#DIV/0!
TRA2B	0	5	#DIV/0!
HBA2	0	5	#DIV/0!
TRIM25	0	5	#DIV/0!
GRB7	0	5	#DIV/0!
ACE2	0	5	#DIV/0!
VDAC3	0	5	#DIV/0!

2nd filtering (Crapome)

1st experience less than 5 times in crapome

User Input	Mapped Gene Symbol	Num of Expt. (found/total)
ITGB4	ITGB4	0
ITGA6	ITGA6	0
CDH1	CDH1	0
MET	MET	0
ITGAV	ITGAV	0
PTPRK	PTPRK	0
PROM1	PROM1	0
CD46	CD46	0
ROR1	ROR1	0
ACE2	ACE2	0
IL1RAP	IL1RAP	0
ITGB6	ITGB6	0
ICAM1	ICAM1	0
MMP15	MMP15	0
F11R	F11R	0
PCDH1	PCDH1	0
NEO1	NEO1	0
F3	F3	0
CDH3	CDH3	0
AXL	AXL	0
DDR1	DDR1	0
TSPAN14	TSPAN14	0
CLDN1	CLDN1	0
PVR	PVR	0
PTPRJ	PTPRJ	0
ADGRG6	ADGRG6	0
NECTIN4	NECTIN4	0
CDCP1	CDCP1	0
PCDHGC3	PCDHGC3	0
LY75	LY75	0
SDK1	SDK1	0
ALCAM	ALCAM	1
PTPRB	PTPRB	1
PTPRF	PTPRF	1
ADGRL2	ADGRL2	1
PLXNA1	PLXNA1	1
DCBLD2	DCBLD2	1
NRP1	NRP1	1
STARD3	STARD3	1
NECTIN2	NECTIN2	1
ITGA1	ITGA1	1
DAG1	DAG1	1
NECTIN3	NECTIN3	1

ERBB2	ERBB2	2
MUC5AC	MUC5AC	2
ADAM10	ADAM10	2
ITGB5	ITGB5	2
IGF1R	IGF1R	2
DPP4	DPP4	2
NCAM1	NCAM1	2
JAG1	JAG1	2
PTK7	PTK7	3
FAT1	FAT1	3
ITGA3	ITGA3	4
ADAM9	ADAM9	4
MYADM	MYADM	5

2nd experience less than 5 times in crapome

User Input	Mapped Gene Symbol	Num of Expt. (found/total)
ITGB4	ITGB4	0
ITGA6	ITGA6	0
ITGAV	ITGAV	0
MET	MET	0
CDH1	CDH1	0
PROM1	PROM1	0
PTPRK	PTPRK	0
PTPRJ	PTPRJ	0
LY75	LY75	0
ITGB6	ITGB6	0
CD46	CD46	0
IL1RAP	IL1RAP	0
CEACAM1	CEACAM1	0
AXL	AXL	0
MST1R	MST1R	0
ICAM1	ICAM1	0
ROR1	ROR1	0
PCDH1	PCDH1	0
NEO1	NEO1	0
F11R	F11R	0
TSPAN14	TSPAN14	0
SPINT1	SPINT1	0
ACE2	ACE2	0
ITGA2	ITGA2	1
PTPRF	PTPRF	1
ALCAM	ALCAM	1
PLXNA1	PLXNA1	1
PTPRB	PTPRB	1
TFPI2	TFPI2	1
ADGRL2	ADGRL2	1
ITGA1	ITGA1	1
CD55	CD55	1
EPHA1	EPHA1	1
DAG1	DAG1	1
ADGRF1	ADGRF1	1
GRB7	GRB7	1
ERBB2	ERBB2	2
ADAM10	ADAM10	2
DPP4	DPP4	2
ITGB5	ITGB5	2
NT5E	NT5E	2
JAG1	JAG1	2
BCAM	BCAM	2

ST14	ST14	2
PTK7	PTK7	3
EPCAM	EPCAM	3
LRP8	LRP8	3
ITGA3	ITGA3	4
ADAM9	ADAM9	4
MYADM	MYADM	5
