# Supplementary material for: Identification of host cell surface proteins inhibiting furin dependent proteolytic processing of viral glycoproteins
Source: Sci Rep. 2025 Jul 15;15:25454. doi: 10.1038/s41598-025-11164-x (PMC12259872; doi:10.1038/s41598-025-11164-x)
Supplement: Supplementary file 8 — Supplementary Information 8. [file 41598_2025_11164_MOESM8_ESM.pdf]

Uncropped Western blot images

Figure 3A

cropped

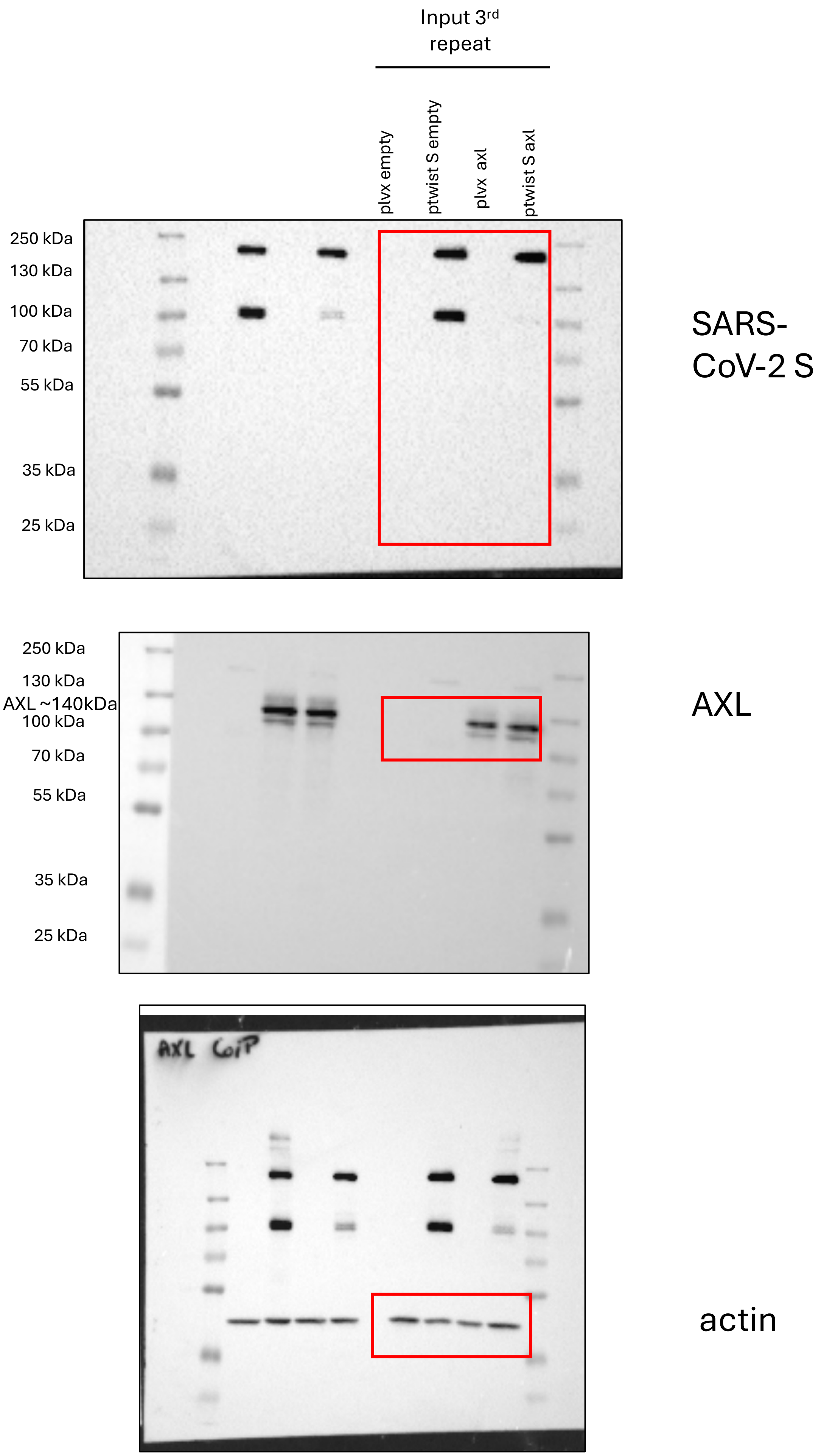

Figure 3B

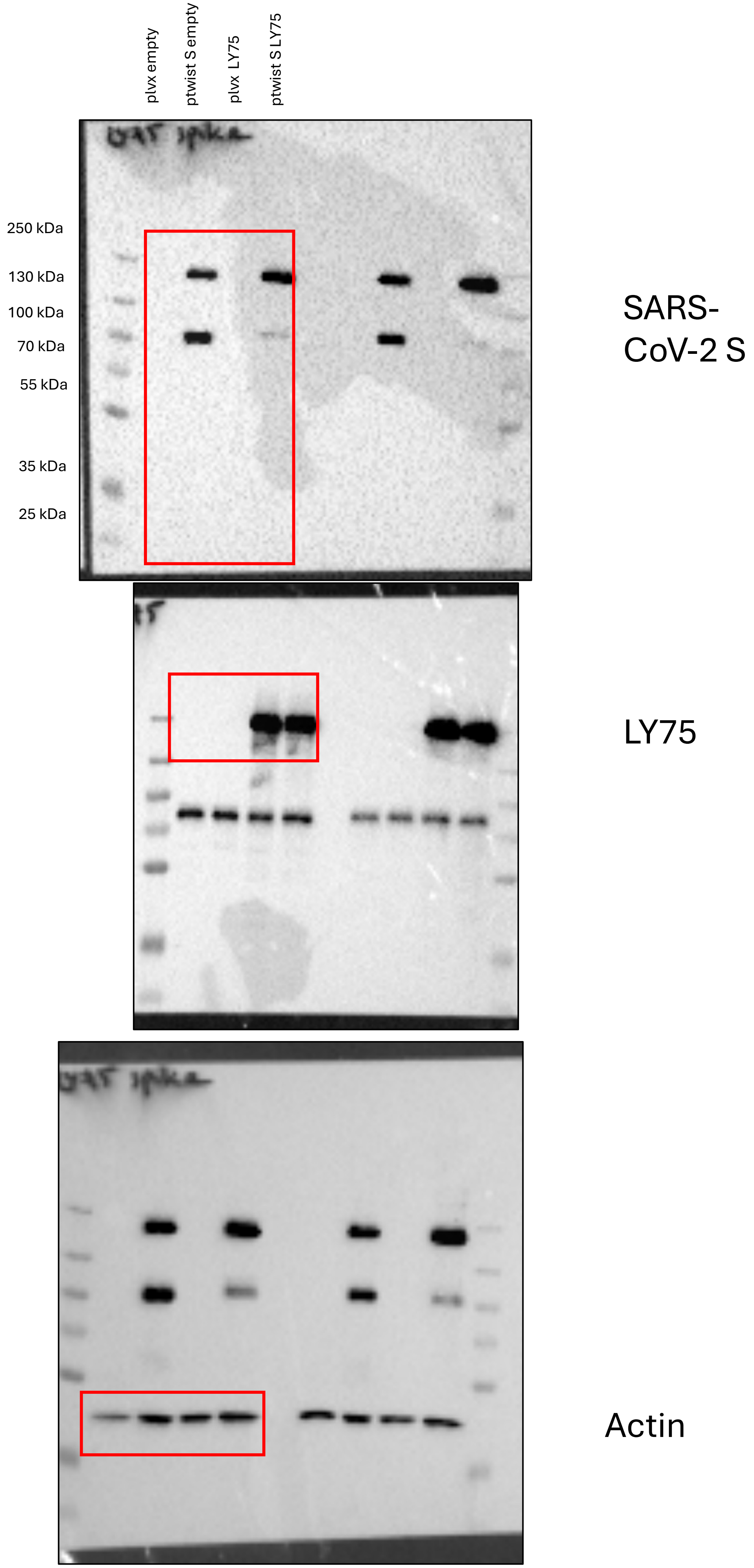

plvx empty
ptwist S empty
plvx LY75
ptwist S LY75
250 kDa
130 kDa
100 kDa
70 kDa
55 kDa
35 kDa
25 kDa
SARS-CoV-2 S
LY75
Actin

Figure 3C

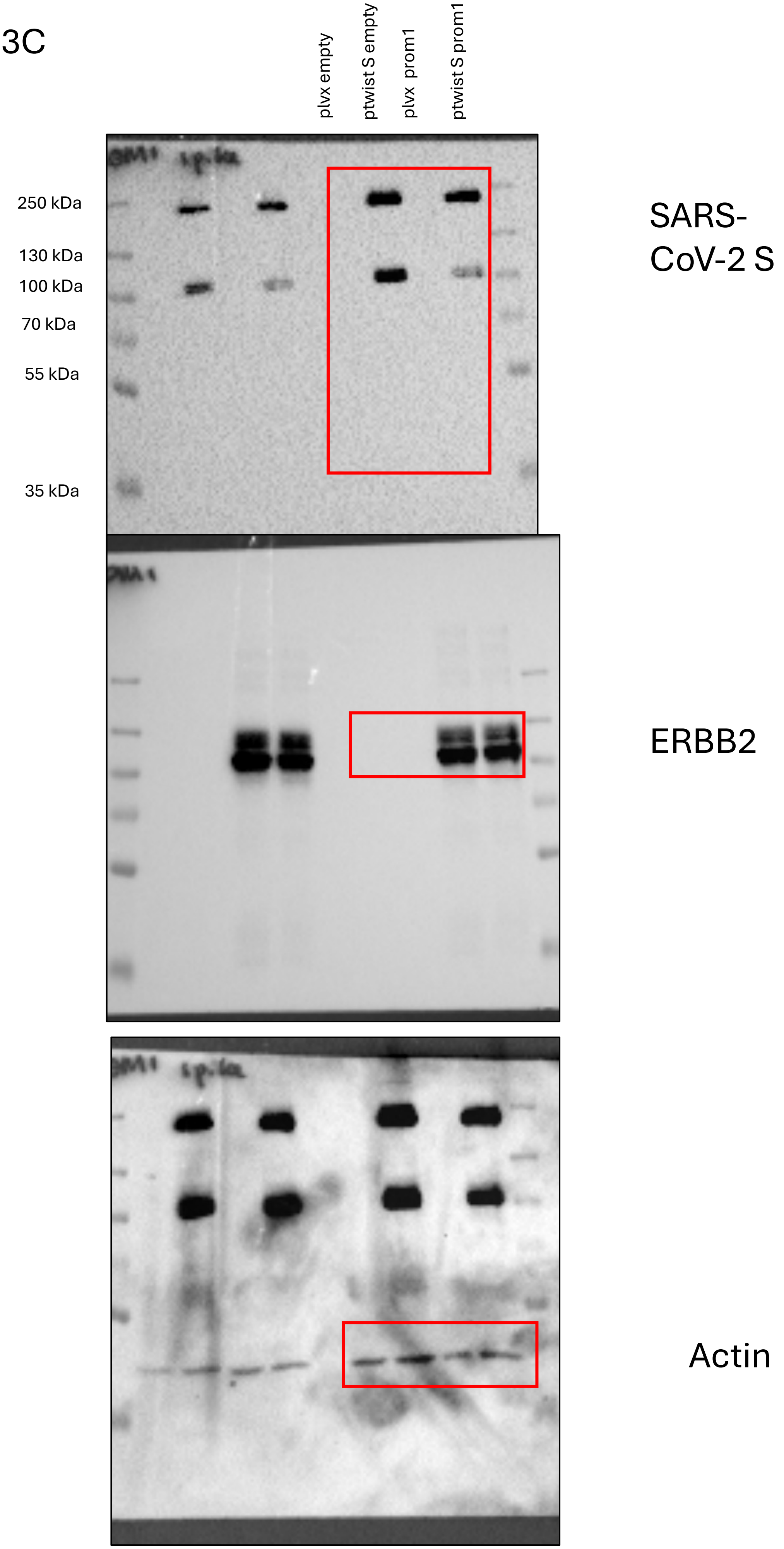
plvx empty
ptwist S empty
plvx prom1
ptwist S prom1
250 kDa
130 kDa
100 kDa
70 kDa
55 kDa
35 kDa
SARS-CoV-2 S
ERBB2
Actin

Figure 3D

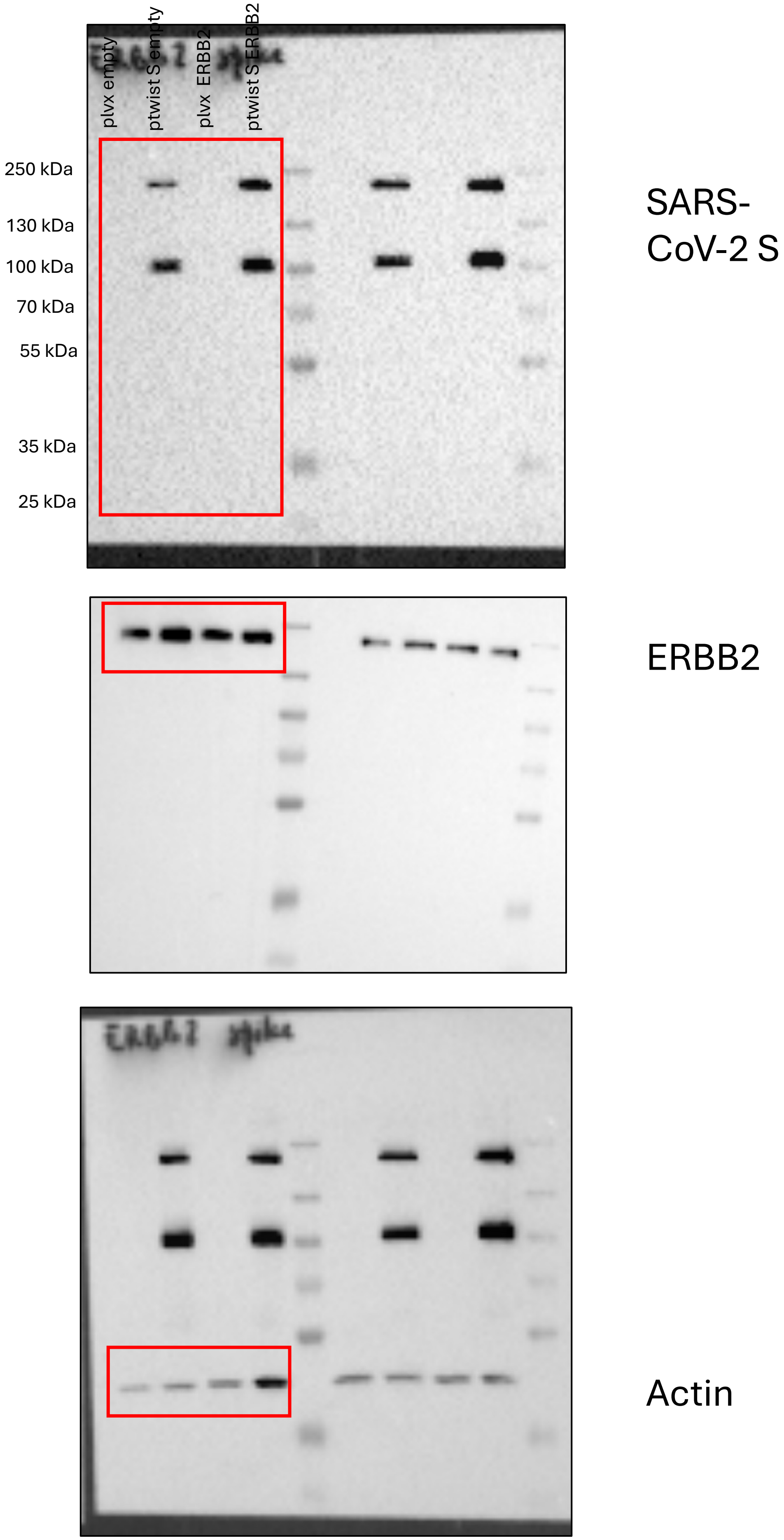

plvx empty
ptwist S empty
plvx ERBB2
ptwist S ERBB2
250 kDa
130 kDa
100 kDa
70 kDa
55 kDa
35 kDa
25 kDa
SARS-CoV-2 S
ERBB2
Actin

Figure 3E

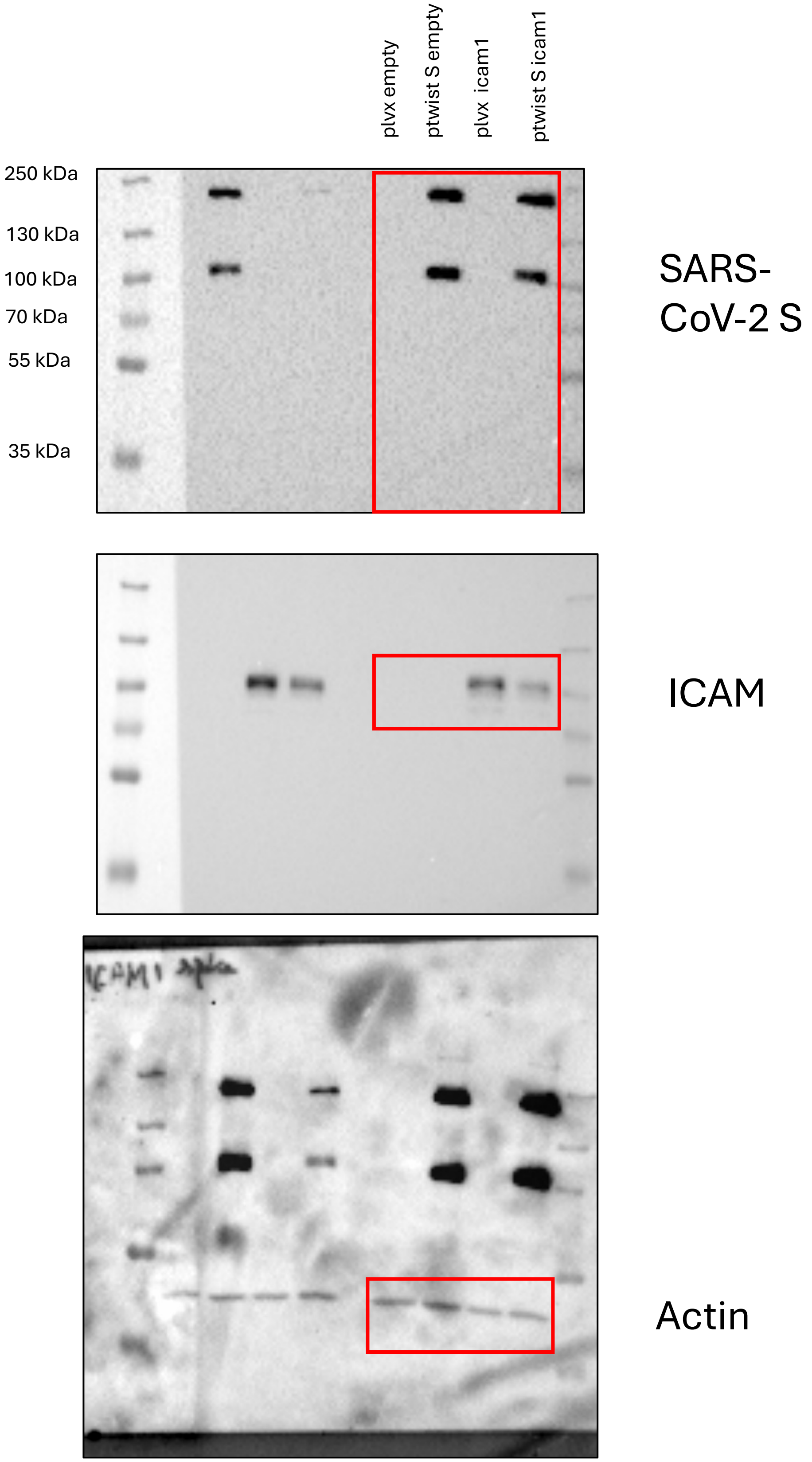
plvx empty
ptwist S empty
plvx icam1
ptwist S icam1
250 kDa
130 kDa
100 kDa
70 kDa
55 kDa
35 kDa
SARS-CoV-2 S
ICAM
Actin

Figure 4B

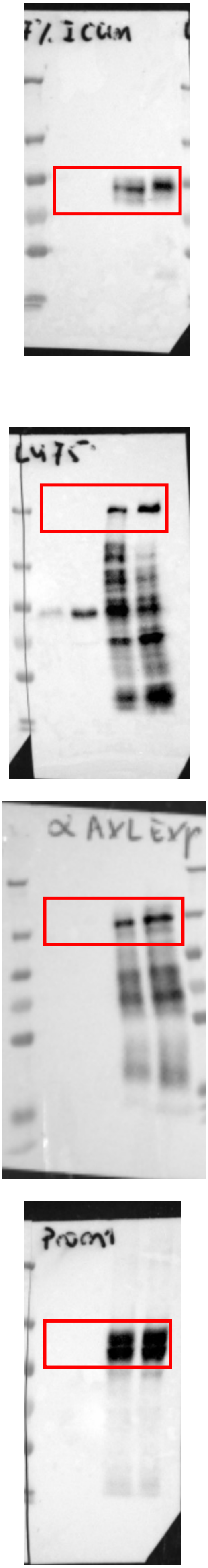

ICAM1

LY75

AXL

PROM1

Figure 5

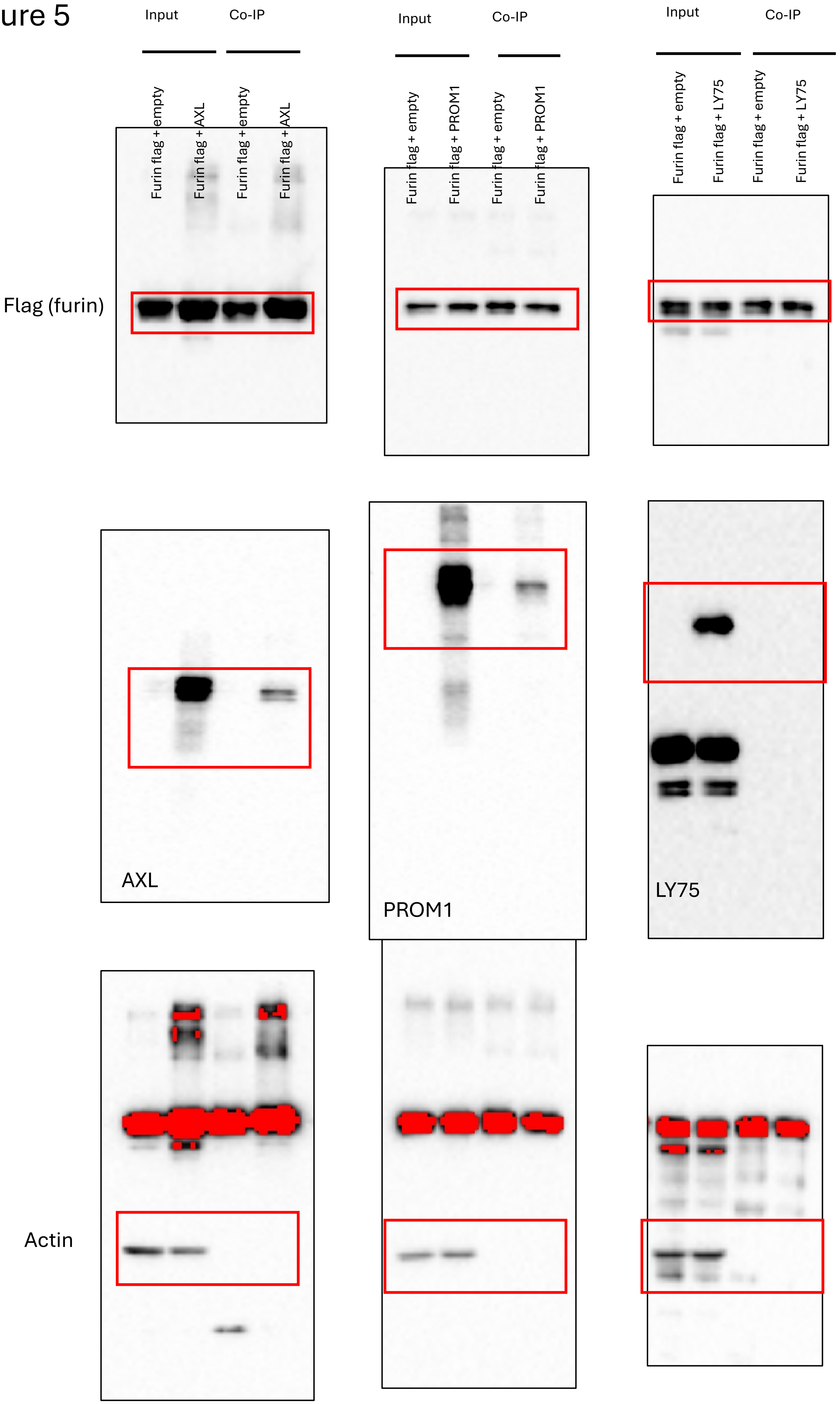
Input
Co-IP
Furin flag + empty
Furin flag + AXL
Furin flag + empty
Furin flag + AXL
Input
Co-IP
Furin flag + empty
Furin flag + PROM1
Furin flag + empty
Furin flag + PROM1
Input
Co-IP
Furin flag + empty
Furin flag + LY75
Furin flag + empty
Furin flag + LY75
Flag (furin)
AXL
PROM1
LY75
Actin

Figure 7A

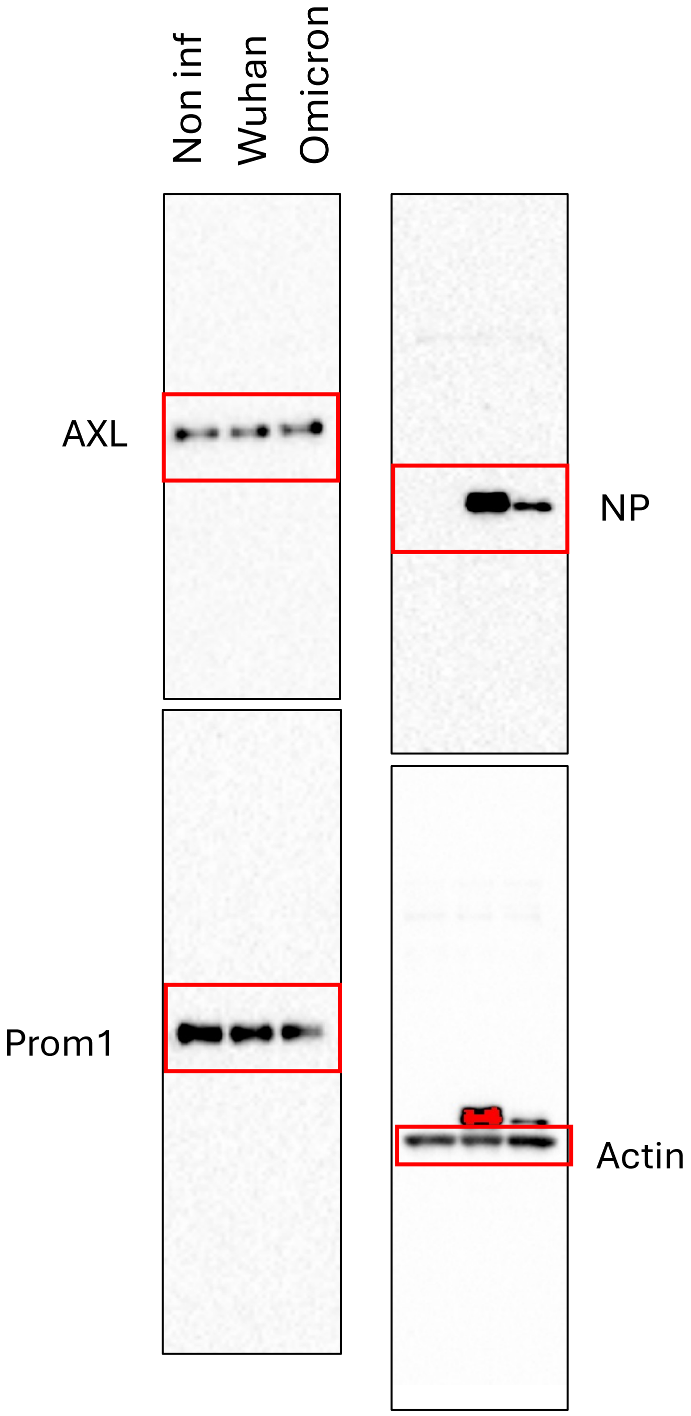

Figure 7D

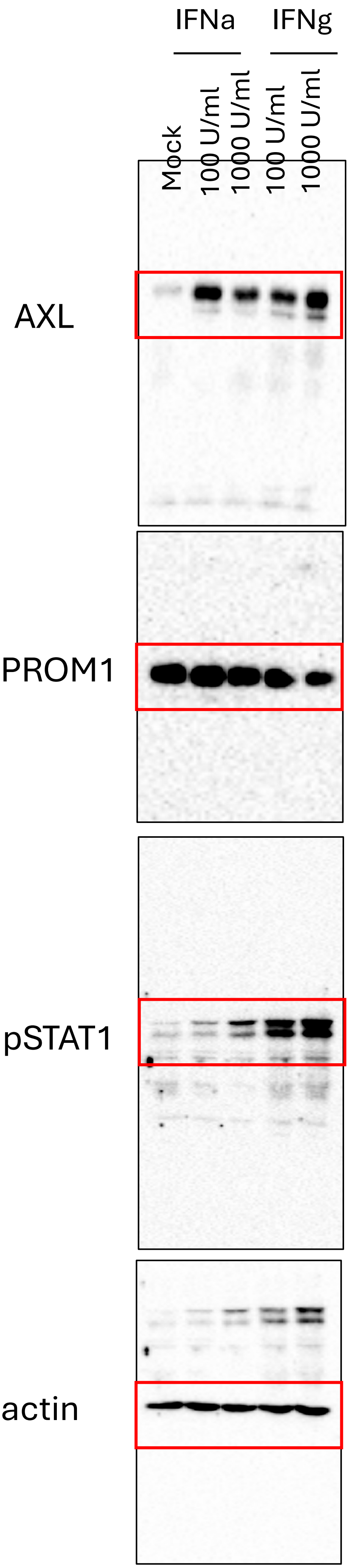
IFNa
IFNg
Mock
100 U/ml
1000 U/ml
100 U/ml
1000 U/ml
AXL
PROM1
pSTAT1
actin

Figure S3

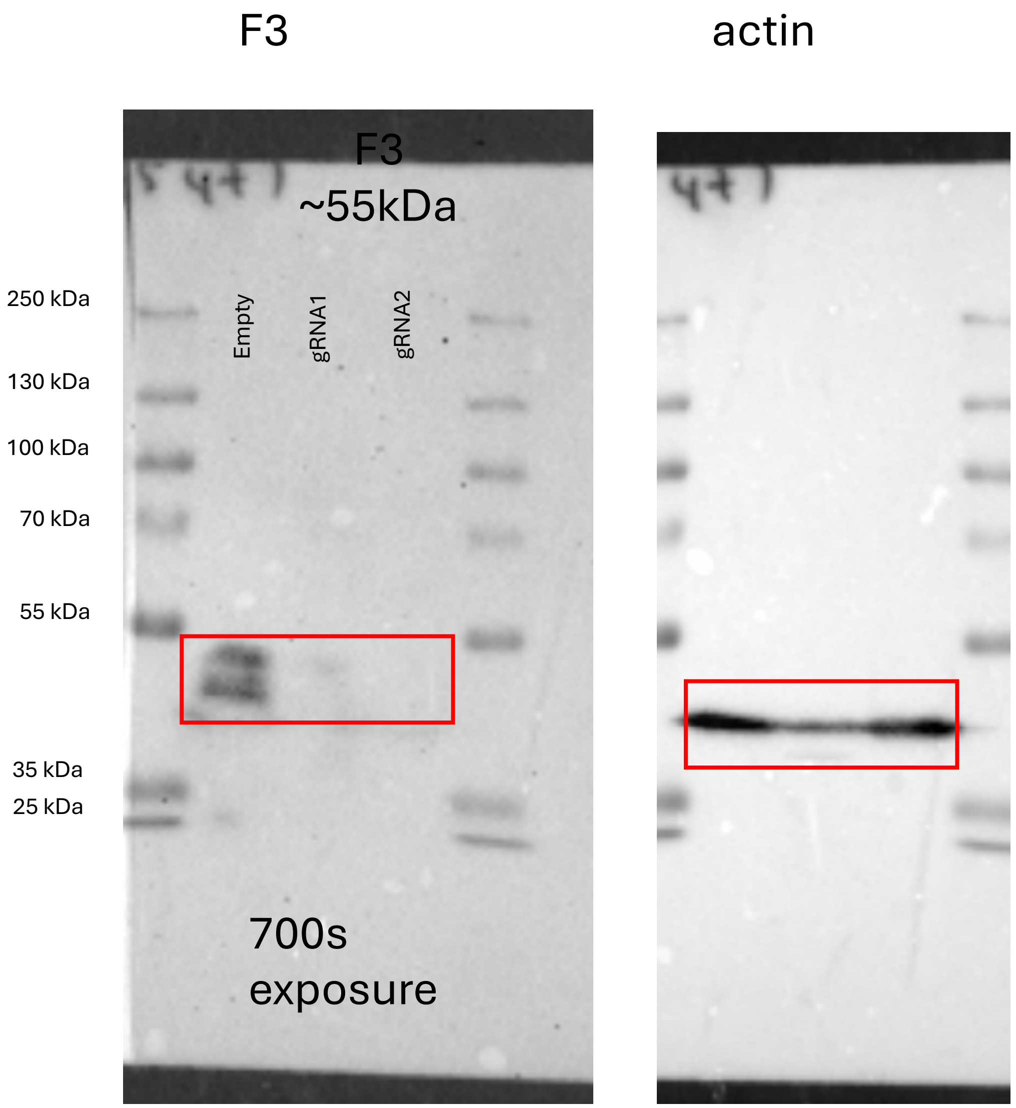
F3
actin
F3
~55kDa
250 kDa
130 kDa
100 kDa
70 kDa
55 kDa
35 kDa
25 kDa
Empty
gRNA1
gRNA2
700s
exposure

Figure S3

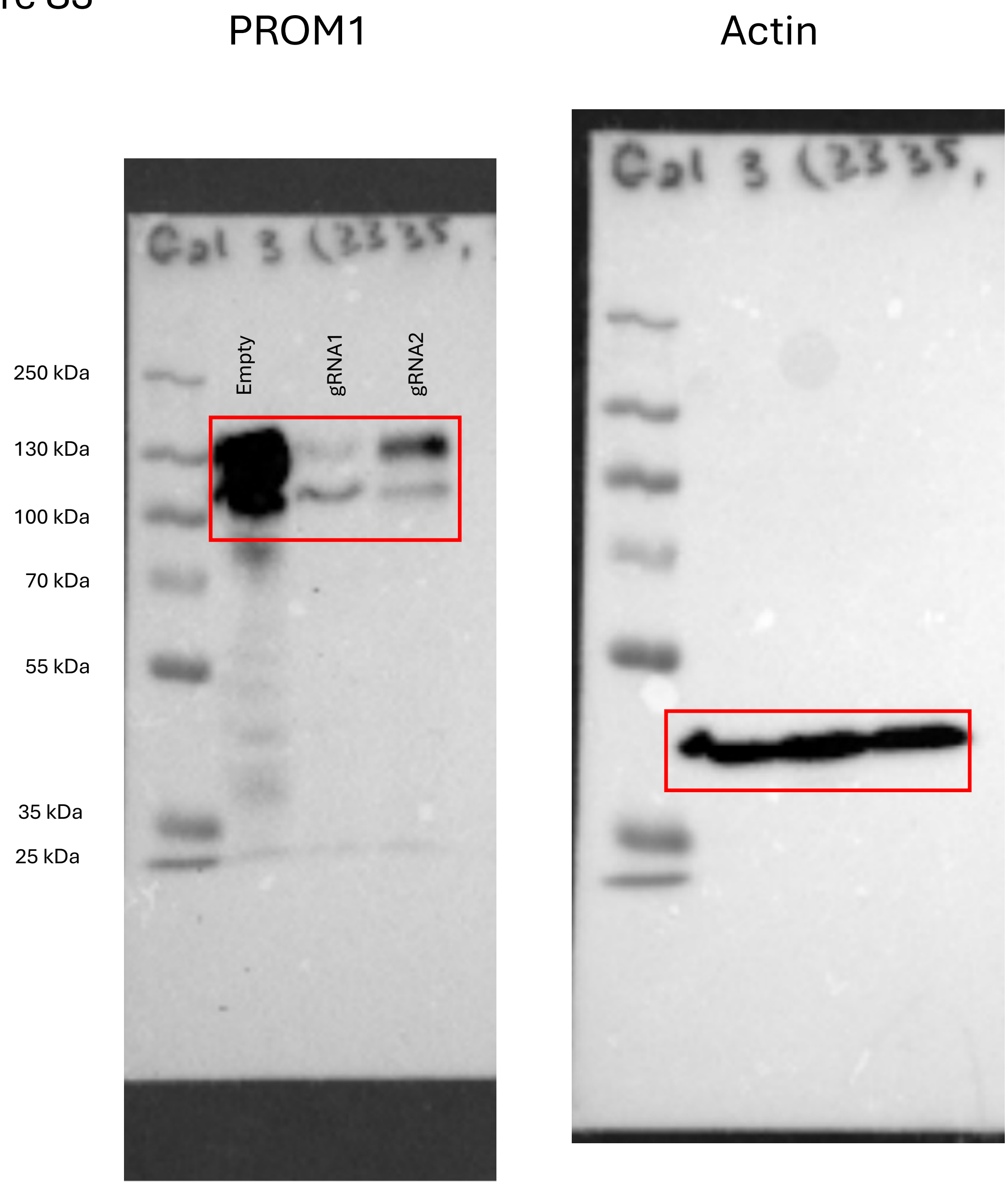
PROM1
Actin
250 kDa
130 kDa
100 kDa
70 kDa
55 kDa
35 kDa
25 kDa
Empty
gRNA1
gRNA2

Figure S3

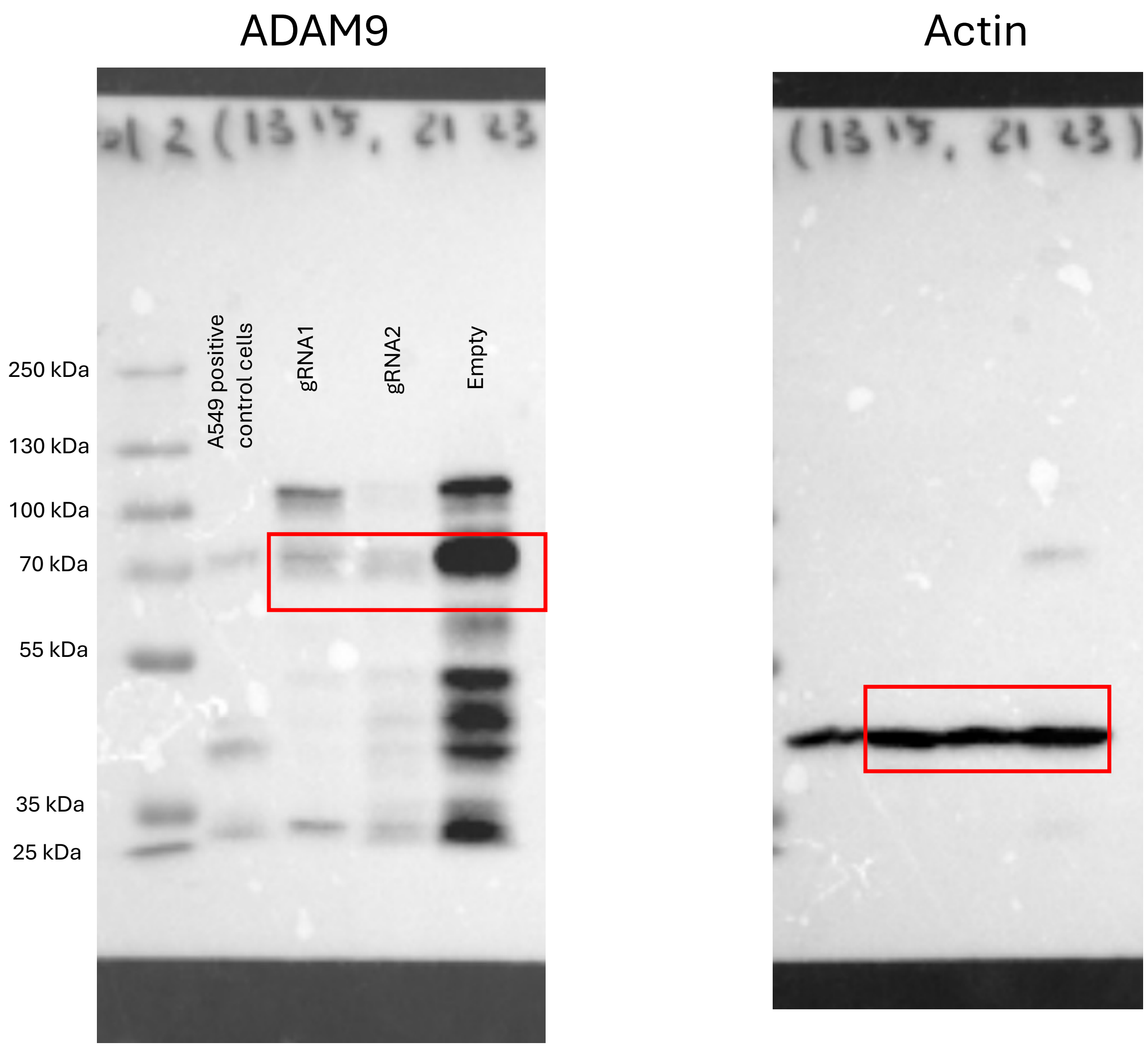

*WB flipped horizontally for figure S3
To maintain logical order of samples

Figure S3

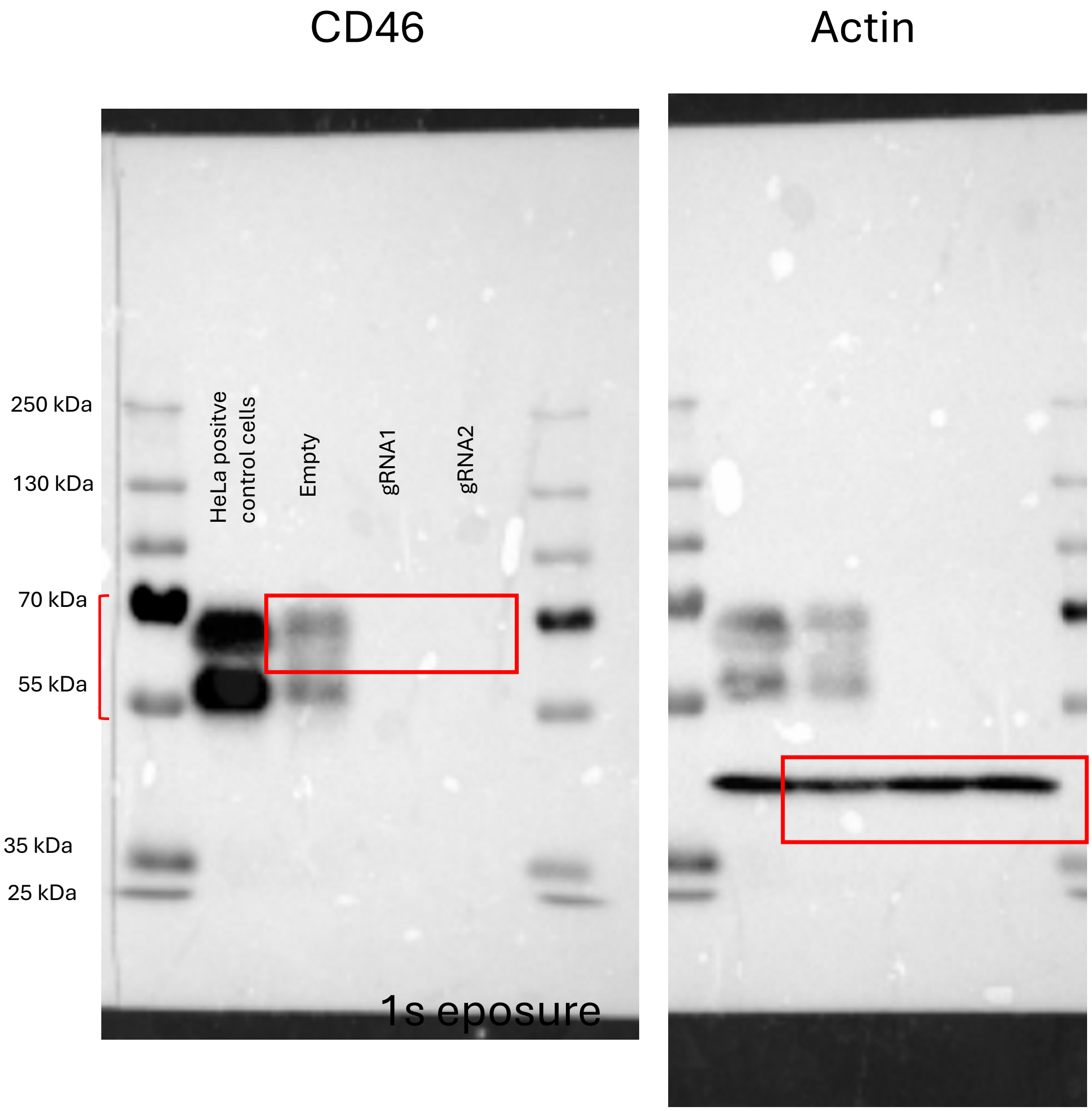
CD46
Actin
250 kDa
130 kDa
70 kDa
55 kDa
35 kDa
25 kDa
HeLa positve
control cells
Empty
gRNA1
gRNA2
1s eposure

Figure S3

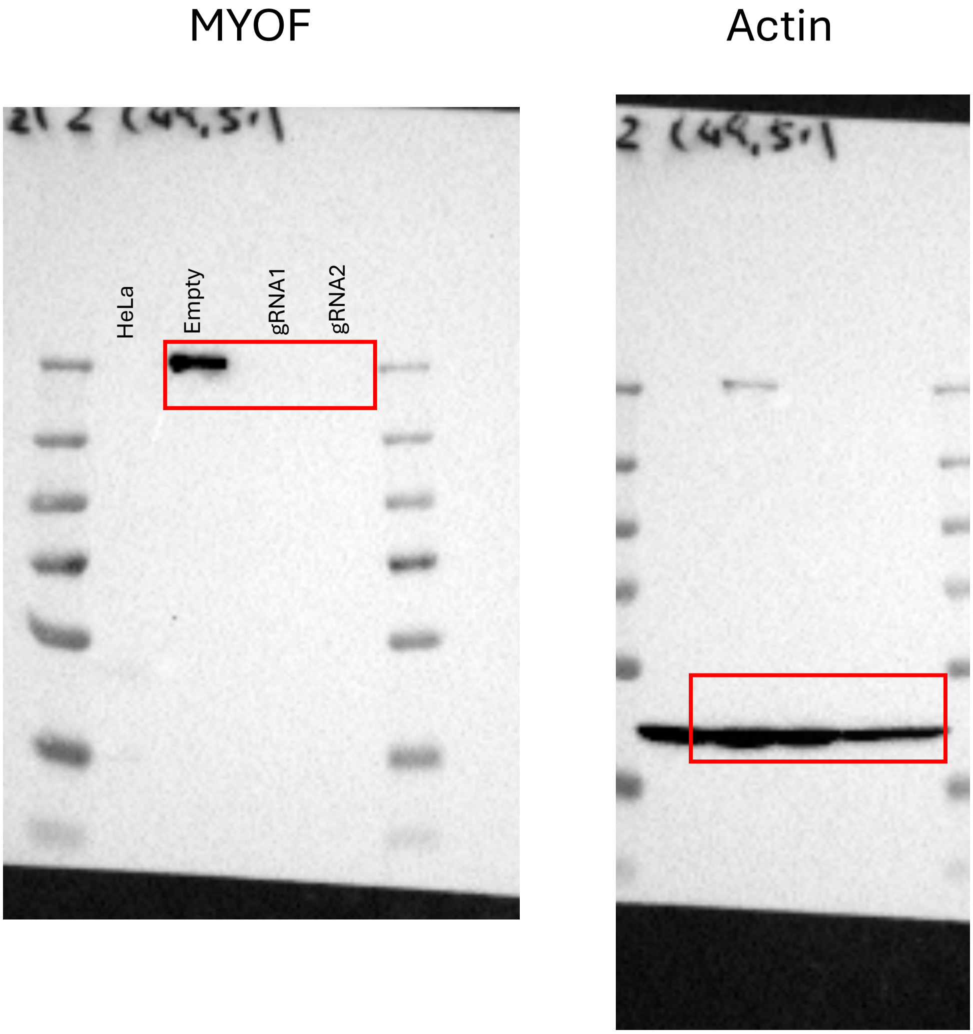

Figure S3

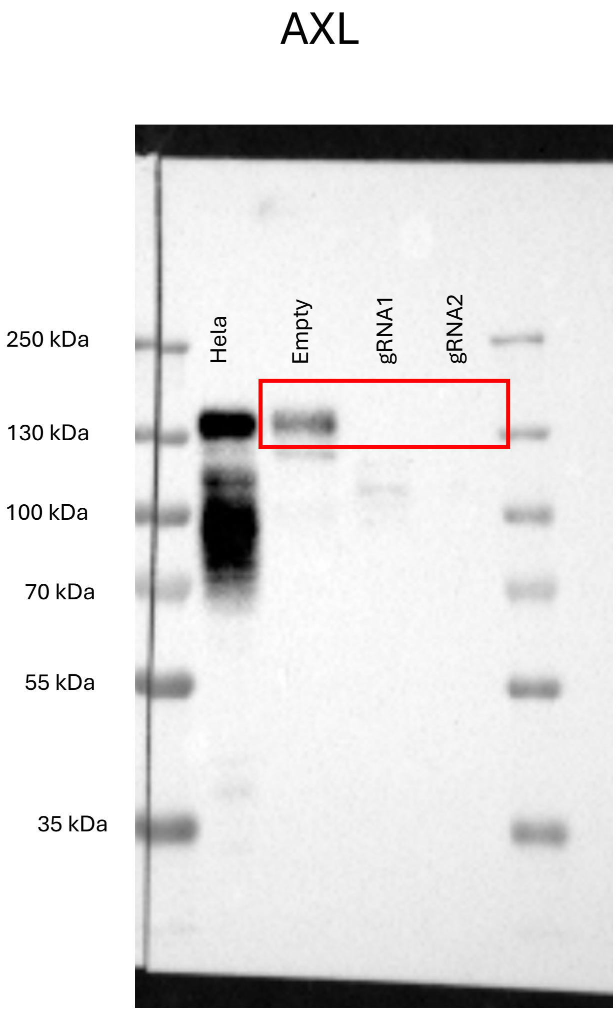
AXL
250 kDa
130 kDa
100 kDa
70 kDa
55 kDa
35 kDa
Hela
Empty
gRNA1
gRNA2

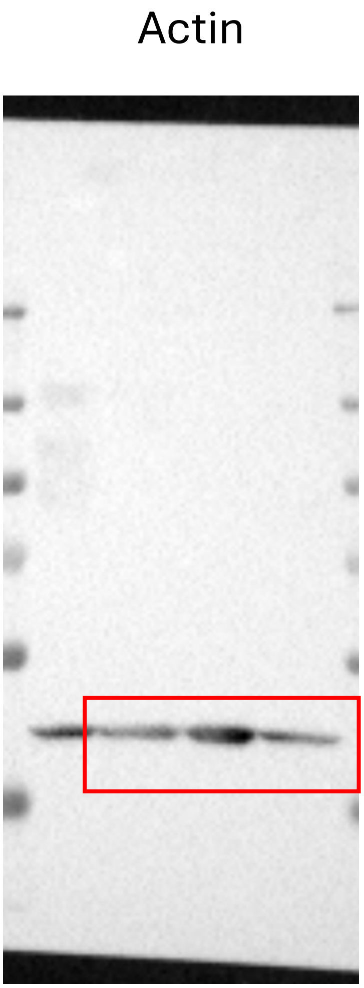
Actin

Figure S3

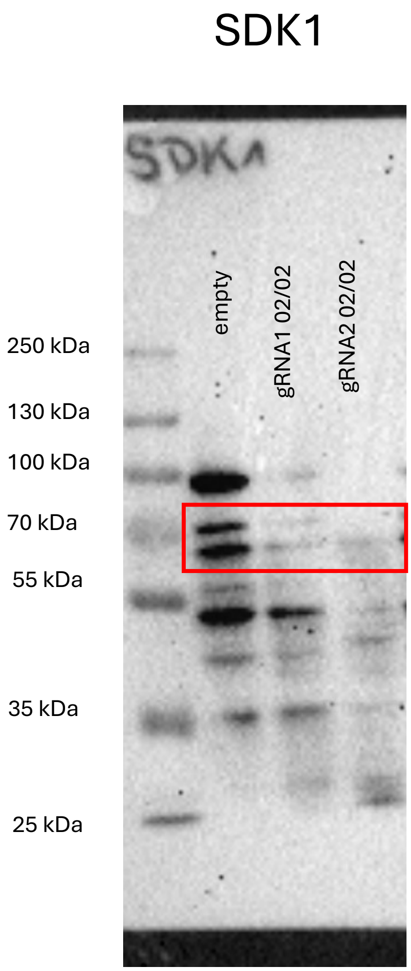

SDK1
250 kDa
130 kDa
100 kDa
70 kDa
55 kDa
35 kDa
25 kDa
empty
gRNA1 02/02
gRNA2 02/02

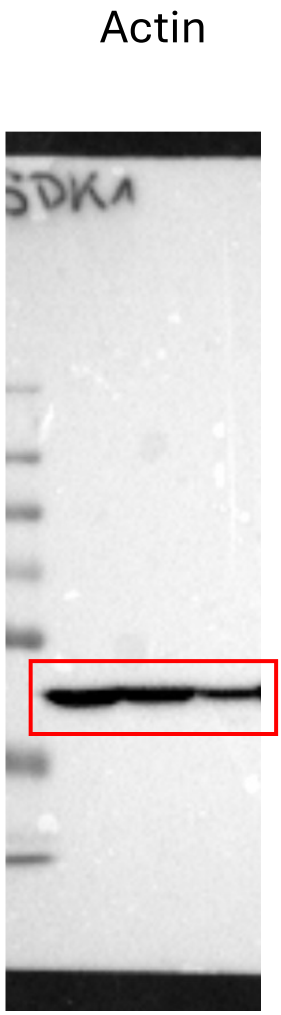

Actin

Figure S3

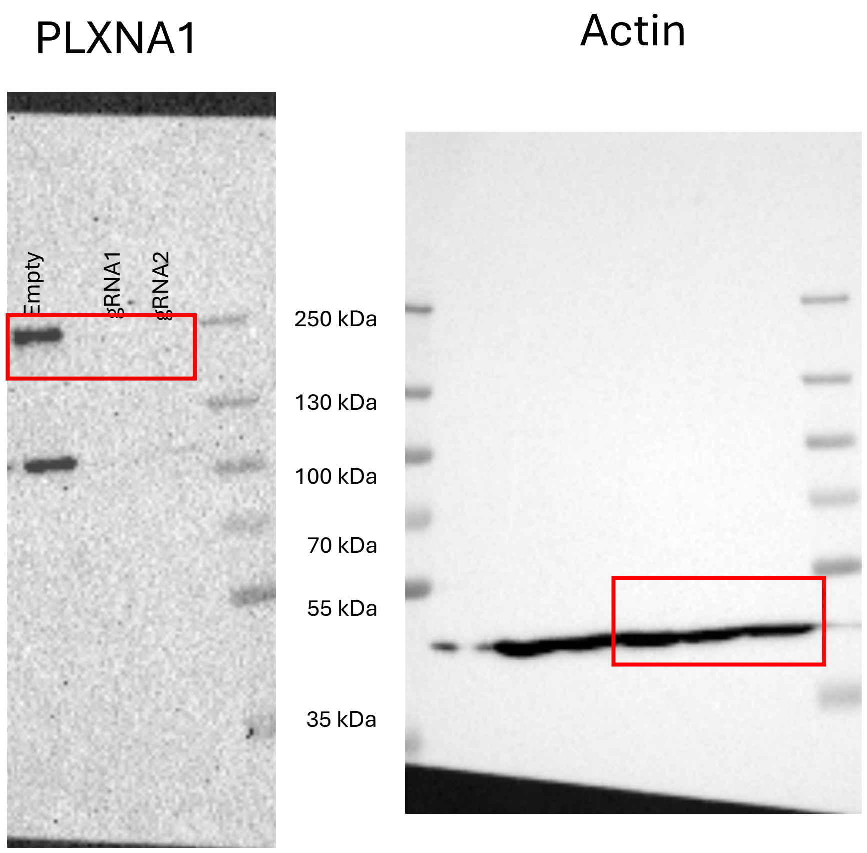

PLXNA1
Actin
Empty
sgRNA1
sgRNA2
250 kDa
130 kDa
100 kDa
70 kDa
55 kDa
35 kDa

Figure S3

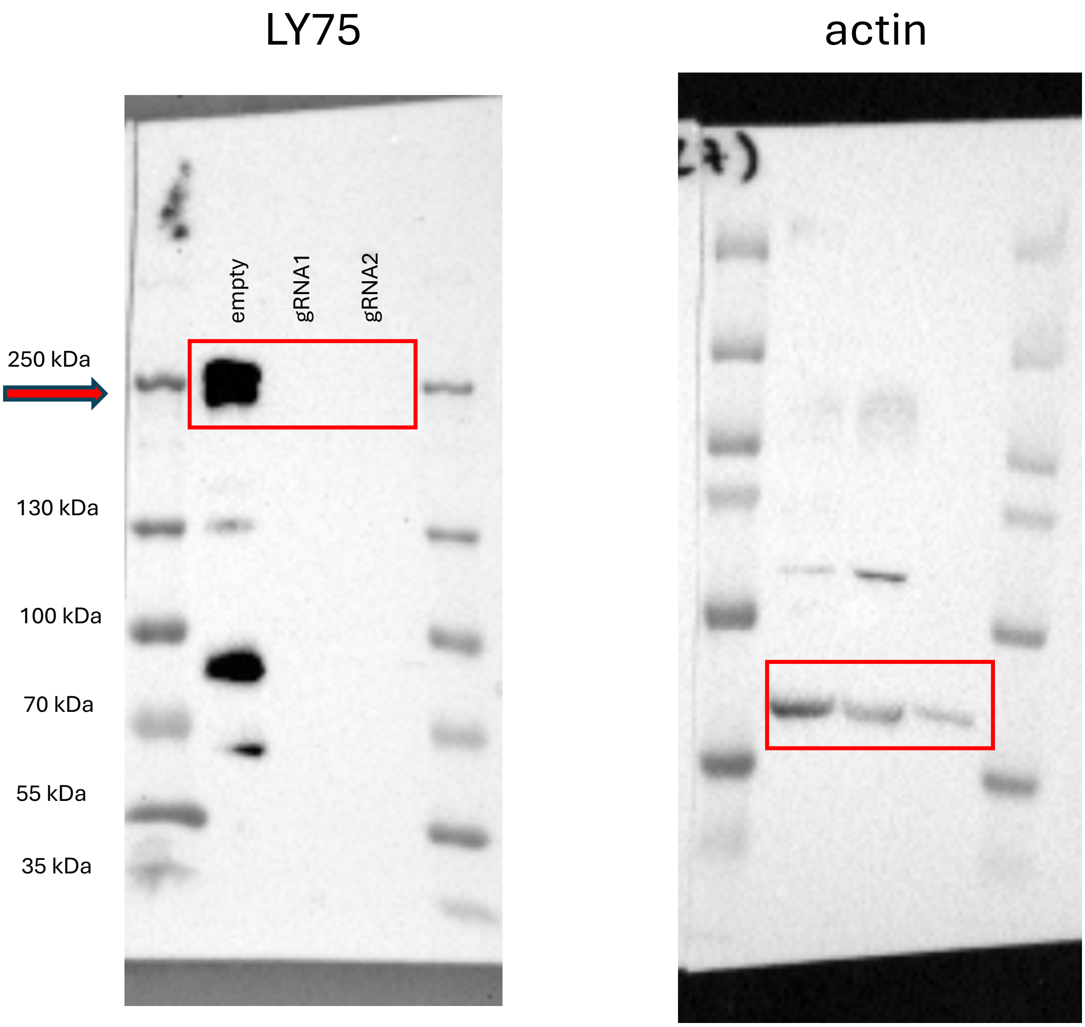
LY75
actin
empty
gRNA1
gRNA2
250 kDa
130 kDa
100 kDa
70 kDa
55 kDa
35 kDa

Figure S3

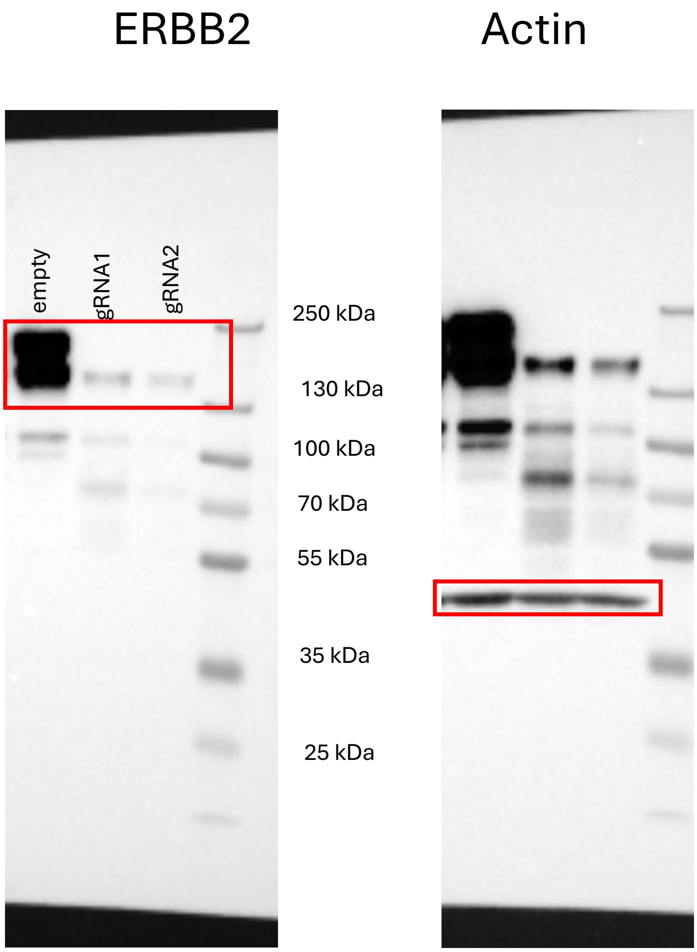

ERBB2
Actin
empty
gRNA1
gRNA2
250 kDa
130 kDa
100 kDa
70 kDa
55 kDa
35 kDa
25 kDa

Figure S3

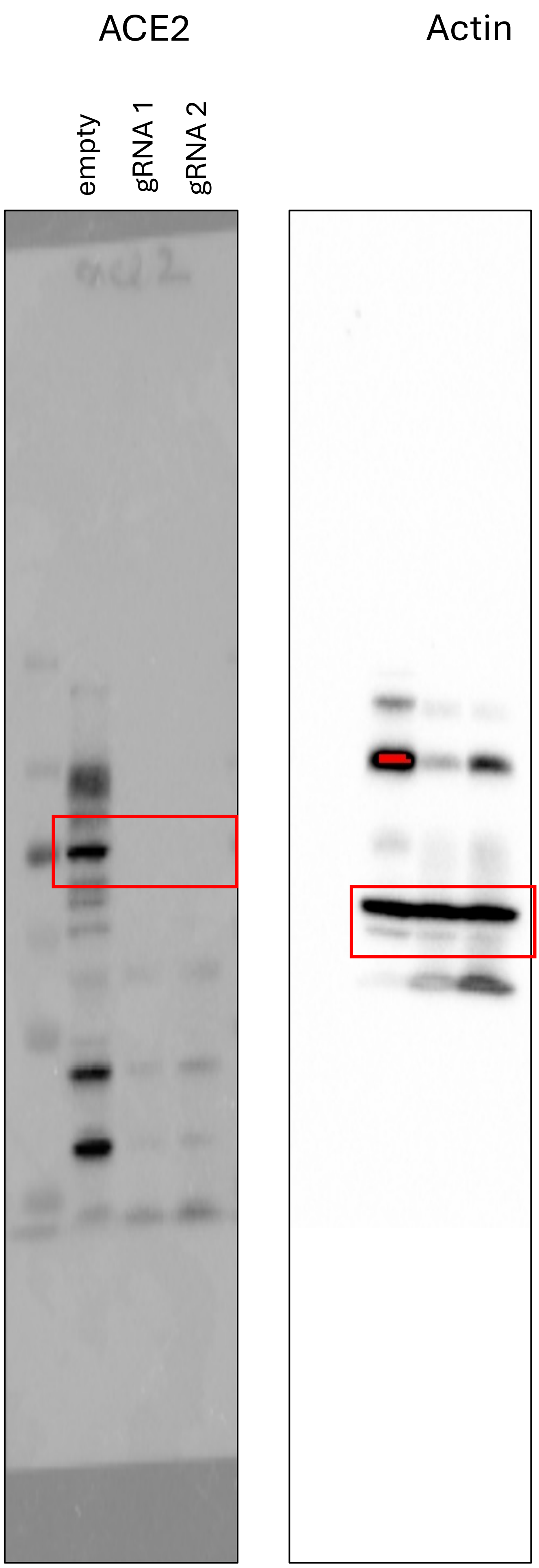

Figure S3

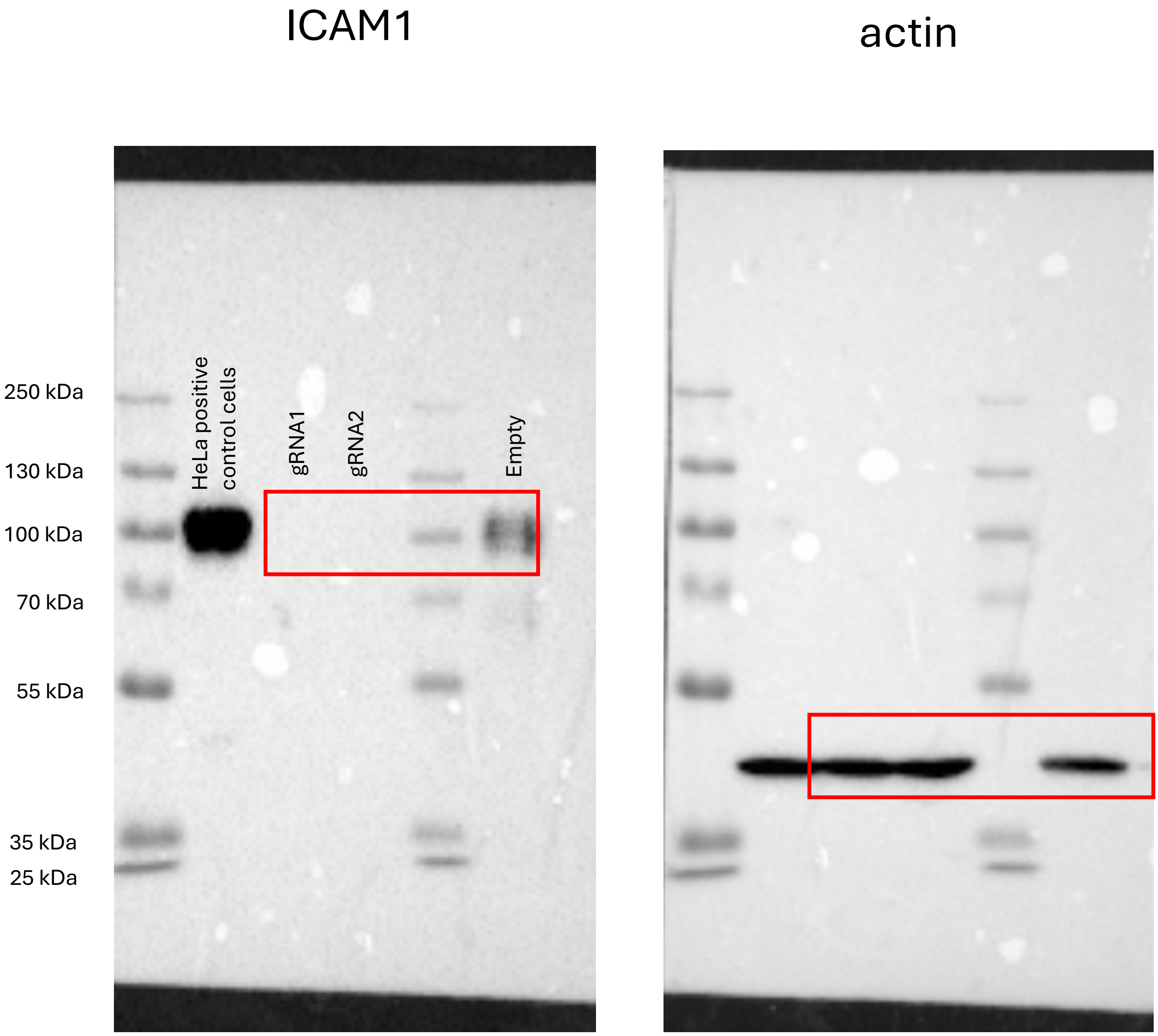

*WB flipped horizontally for figure S3
To maintain logical order of samples
